# A Large Polysaccharide Produced by *Helicobacter hepaticus* Induces an Anti-inflammatory Gene Signature in Macrophages

**DOI:** 10.1016/j.chom.2017.11.002

**Published:** 2017-12-13

**Authors:** Camille Danne, Grigory Ryzhakov, Maria Martínez-López, Nicholas Edward Ilott, Fanny Franchini, Fiona Cuskin, Elisabeth C. Lowe, Samuel J. Bullers, J. Simon C. Arthur, Fiona Powrie

**Affiliations:** 1 Kennedy Institute of Rheumatology, University of Oxford, Oxford, UK; 2 Immunobiology Laboratory, Fundación Centro Nacional de Investigaciones Cardiovasculares “Carlos III” (CNIC), Melchor Fernández Almagro 3, Madrid, Spain; 3 Institute for Cell and Molecular Biosciences, Medical School Newcastle University, Newcastle upon Tyne, UK; 4 Division of Cell Signaling and Immunology, School of Life Sciences, University of Dundee, Dundee, UK

**Keywords:** inflammatory bowel disease, host-microbe interactions, mutualism, *Helicobacter hepaticus*, macrophage, anti-inflammatory gene signature, polysaccharide, TLR2, CREB, MSK1/2

## Abstract

Interactions between the host and its microbiota are of mutual benefit and promote health. Complex molecular pathways underlie this dialog, but the identity of microbe-derived molecules that mediate the mutualistic state remains elusive. *Helicobacter hepaticus* is a member of the mouse intestinal microbiota that is tolerated by the host. In the absence of an intact IL-10 signaling, *H. hepaticus* induces an IL-23-driven inflammatory response in the intestine. Here we investigate the interactions between *H. hepaticus* and host immune cells that may promote mutualism, and the microbe-derived molecule(s) involved. Our results show that *H. hepaticus* triggers early IL-10 induction in intestinal macrophages and produces a large soluble polysaccharide that activates a specific MSK/CREB-dependent anti-inflammatory and repair gene signature via the receptor TLR2. These data identify a host-bacterial interaction that promotes mutualistic mechanisms at the intestinal interface. Further understanding of this pathway may provide novel prevention and treatment strategies for inflammatory bowel disease.

## Highlights

• *Helicobacter hepaticus* (*Hh*) activates a specific anti-inflammatory program in macrophages
• This activity is driven by an *Hh* polysaccharide inducing high IL-10/IL-6 ratio in BMDMs
• The polysaccharide-specific response is dependent on the TLR2/MSK/CREB pathway

## Graphical Abstract

**Figure:**
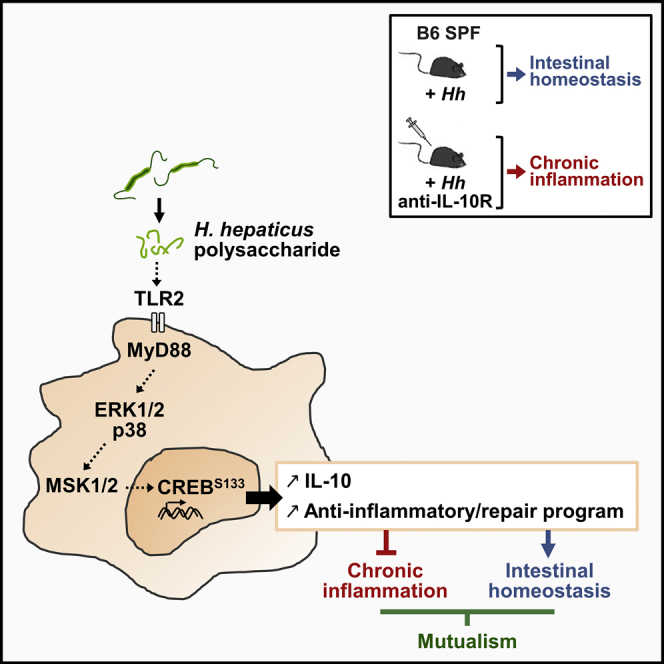

## Teaser

Host-microbiota interactions are of mutual benefit, and chronic intestinal inflammation develops when this dialog is altered. Danne et al. identified a polysaccharide produced by *Helicobacter hepaticus* that induces a specific anti-inflammatory and repair program in macrophages by activating the TLR2/MSK/CREB pathway. Further understanding may provide prevention and treatment strategies for IBD.

## Introduction

Shaped by a long history of co-evolution, the relationship between mammalian hosts and their intestinal commensal bacteria is of mutual benefit and promotes health. Complex molecular pathways underlie this dialog; however, the identity of microbe-derived molecules that contribute to the mutualistic state remain elusive. In inflammatory bowel disease (IBD), maladaptation of the host-microbe interface results in aberrant inflammatory responses to the intestinal microbiota. Recent evidence suggests that a complex interplay of host genetic, environmental, and microbial factors contributes to disease development (Kaser et al., 2010). Studies both in mouse models and in human disease have highlighted the role of appropriate intestinal epithelial barrier function, host defense, and immune regulation for maintaining intestinal homeostasis (Maloy and Powrie, 2011).

Infection of mice with the mouse pathogen *Helicobacter hepaticus* has provided important insights into host-microbe interactions in the gut. *H. hepaticus* inhabits the lower intestine, primarily the caecum, but does not induce immune pathology in normal mice (Kullberg et al., 1998). However, infection of genetically susceptible T and B cell-deficient 129SvEv*Rag*^−/−^ mice results in colitis (Erdman et al., 2003, Maloy et al., 2003), and *H. hepaticus* exacerbates T cell transfer colitis in C.B17 severe combined immunodeficient mice (Cahill et al., 1997). Appropriate host immune regulatory responses are crucial, as *H. hepaticus* infection of lymphocyte-replete mice with genetic- or pharmacological-induced deficiencies of the interleukin-10/interleukin-10 receptor (IL-10/IL-10R) pathway also results in colitis and typhlitis (Kullberg et al., 1998, Kullberg et al., 2006, Morrison et al., 2013). Under these circumstances, there is an aberrant IL-23-driven inflammatory response in the intestine with accumulation of pro-inflammatory granulocytes and monocytes that contributes to disease pathology (Arnold et al., 2016, Griseri et al., 2012, Hue et al., 2006, Krausgruber et al., 2016). Early studies showed that infection with *H. hepaticus* induces colonic regulatory T cells that prevent inflammation in an IL-10-dependent manner (Kullberg et al., 2002) and, more recently, that large amounts of IL-23 impede this response (Schiering et al., 2014). Relying both on a bacterial trigger and on an immune defect, *H. hepaticus-*induced colitis in the presence of IL-10/IL-10R pathway deficiency shares many features of human IBD. Indeed, mutations in *Il10* or *Il10R* result in severe early-onset forms of IBD (Glocker et al., 2009, Kotlarz et al., 2012, Uhlig et al., 2014), indicating that IL-10 signaling is critical to prevent colitis both in humans and in mice. To date, little is known about the interaction of *H. hepaticus* with the innate immune compartment and its capacity to induce IL-10 production by these cells.

Strategically located at the mucosal barrier, intestinal lamina propria-resident macrophages function as immune sentinels. Essential for maintaining homeostatic responses, they are both sensors and interpreters of the intestinal tissue microenvironment, which comprises microbes and their products, and of immune mediators such as cytokines and chemokines (Ginhoux et al., 2016). The loss of IL-10R in tissue-resident macrophages results in spontaneous colitis, highlighting the importance of the macrophage response to IL-10 (Zigmond et al., 2014). Microbial products from both commensals and pathogens are recognized by pattern recognition receptors (PRRs), including Toll-like receptors (TLRs). The activation of PRRs triggers the production of pro-inflammatory mediators, such as IL-6 and tumor necrosis factor-α (TNFα), via several signaling pathways including the transcription factor NF-κB (Cohen, 2014). To prevent chronic inflammation and tissue damage, PRRs also activate anti-inflammatory signals, such as IL-10, together with negative feedback mechanisms, including the transcriptional repressor cAMP response element-binding protein (CREB) (Wen et al., 2010). However, the signaling mechanisms employed by particular gut inhabitants to actively promote intestinal homeostasis are poorly understood, as is their interaction with intestinal macrophages.

Here, we have investigated the interactions between *H. hepaticus* and macrophages that may promote tolerance and mutualism. Our results show that *H. hepaticus* induces an early IL-10 response by intestinal macrophages and produces a large soluble polysaccharide that activates a specific MSK/CREB-dependent anti-inflammatory and repair gene signature via the receptor TLR2.

## Results

### *H. hepaticus* Induces IL-10 in Gut-Resident Macrophages

**Figure 1:**
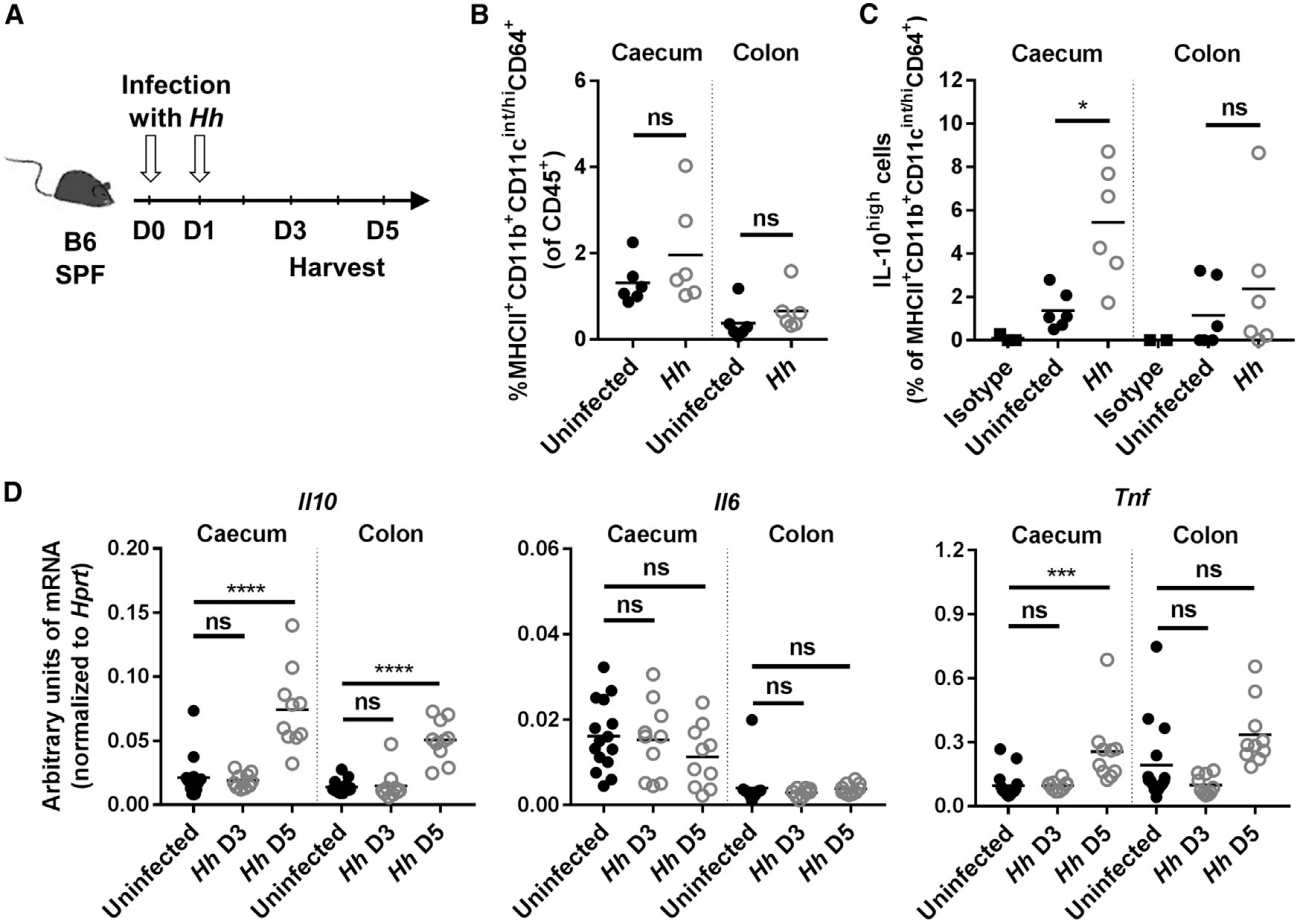
*H. hepaticus* Induces IL-10 in Gut-Resident Macrophages (A–D) SPF WT mice were infected with *Helicobacter hepaticus* (*Hh*) for 3 or 5 days. (A) Experimental design. (B and C) FACS analysis of caecum and colon LPL after 3 days of infection with *Hh*. (B) Frequency of resident macrophages among total CD45^+^ cells. (C) Frequency of IL-10^high^ cells among resident macrophages, using an anti-mouse IL-10 antibody or its isotype control. (D) Expression level of *Il10*, *Il6*, and *Tnf* mRNA in caecum or colon tissue after 3 or 5 days of infection with *Hh*. Each symbol represents an individual mouse (two to three independent experiments). Mann-Whitney test (B and C) or one-way ANOVA and Tukey’s multiple comparisons test (D), p < 0.05. See also Figure S1.

As blockade of IL-10 signaling induces colitis in mice colonized with *H. hepaticus* (Kullberg et al., 2006, Morrison et al., 2013), we first assessed whether *H. hepaticus* interacts directly with the innate immune compartment within the intestinal mucosa to trigger IL-10 production. In order to investigate early responses to *H. hepaticus* at the cellular level, lamina propria leukocytes (LPL) from the colonic and caecal mucosa were isolated from Specific Pathogen Free (SPF) mice 3 days after infection with *H. hepaticus* strain ATCC51449 (Figure 1A). Flow cytometric analysis revealed that the frequency of CD4^+^ IL-10^high^ T cells did not change at this time point (Figure S1A). Similarly, the frequency of MHCII^+^CD11b^+^CD11c^int/high^CD64^+^ resident macrophages among total CD45^+^ cells was unaffected by *H. hepaticus* infection (Figure 1B). However, the percentage of IL-10^high^ resident macrophages increased significantly in the caecum following *H. hepaticus* colonization (Figures 1C and S1B). In whole tissue, no difference in levels of *Il10* mRNA could be detected 3 days following infection (Figure 1D). However, there was a marked increase in the amount of *Il10* mRNA 5 days after infection in both caecum and colon, whereas mRNA levels of the pro-inflammatory cytokines IL-6 and TNFα were unchanged or moderately increased, respectively (Figure 1D). These data indicate that *H. hepaticus* selectively promotes IL-10 production without a corresponding increase in pro-inflammatory cytokines from intestinal macrophages.

### *H. hepaticus* Produces a Large Soluble Polysaccharide that Can Induce IL-10 Production by Macrophages

**Figure 2:**
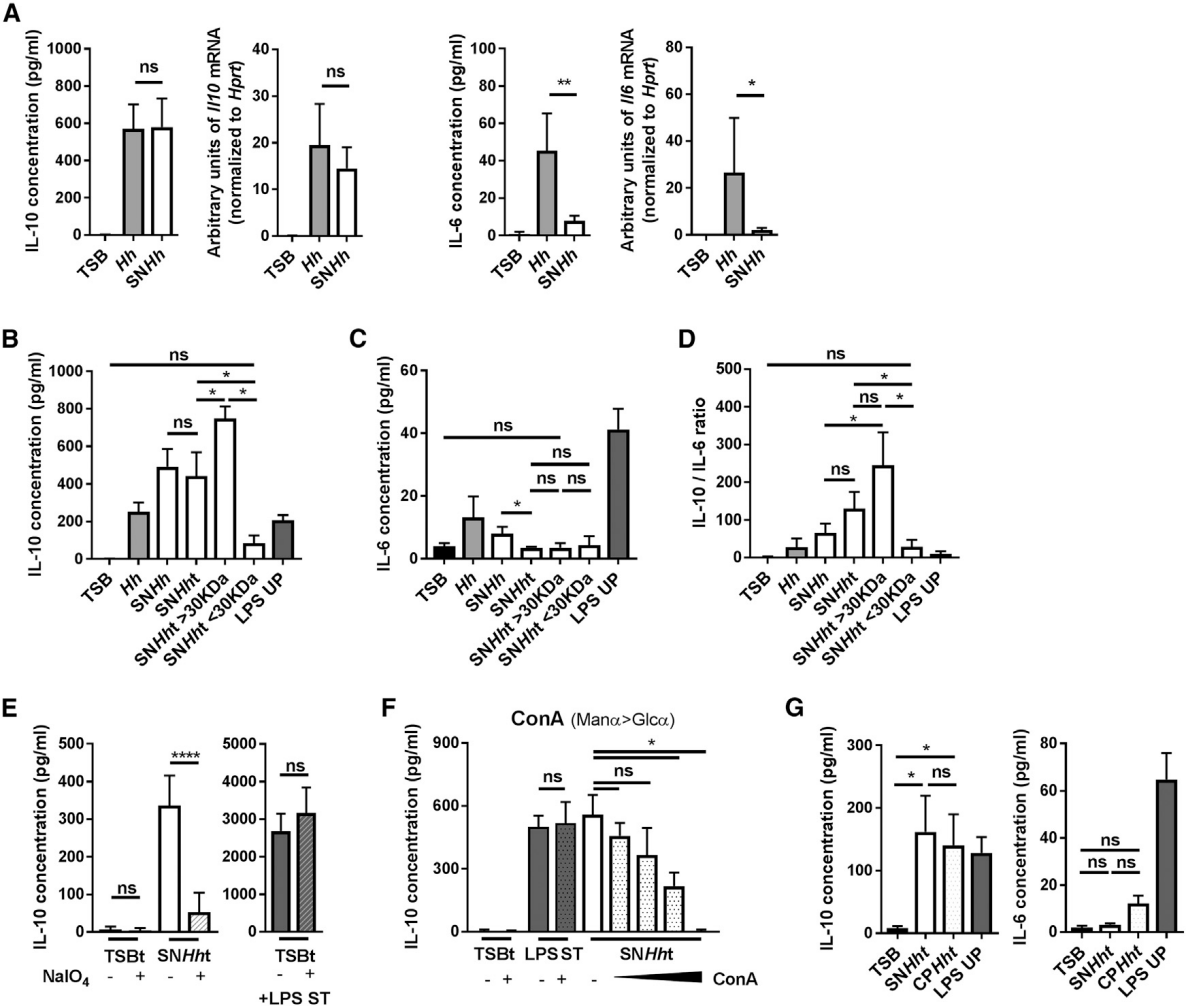
*H. hepaticus* Produces a Large Soluble Polysaccharide-Inducing IL-10 Production in Macrophages (A–G) M-CSF-differentiated BMDMs were stimulated for 3 hr with different culture fractions of *H. hepaticus*. (A) mRNA and protein induction of the cytokines IL-10 and IL-6 after stimulation with control medium (TSB), *H. hepaticus* whole bacteria (*Hh*), or *H. hepaticus*-filtered cultured supernatant (SN*Hh*). (B–D) Induction of (B) IL-10, (C) IL-6, and (D) IL-10/IL-6 ratio after 3 hr stimulation with SN*Hh* treated with enzymes and heat (SN*Hh*t), SN*Hh*t fractionated by size (SN*Hh*t > 30 kDa and SN*Hh*t < 30 kDa), and LPS UP. (E) Induction of IL-10 after stimulation with TSBt or SN*Hh*t, treated with buffer (−) or sodium metaperiodate (+, NaIO_4_). Stimulation with TSBt ± NaIO_4_ and LPS ST is used as a positive control for IL-10 production by BMDMs. (F) Induction of IL-10 after stimulation with TSBt, LPS ST, SN*Hh*t, or SN*Hh*t depleted using increasing concentrations of ConA-lectin beads (0.8%, 1.5%, 3%, and 6% v/v). (G) Induction of IL-10 and IL-6 after stimulation with SN*Hh*t crude polysaccharide fraction extracted by a cold-ethanol precipitation method (CP*Hh*t). Data from three independent experiments. Mann-Whitney test, p < 0.05. Mean ± SD. LPS UP, LPS ultrapure from *E. coli* O111:B4; LPS ST, LPS standard from *E. coli* O55:B5. See also Figure S2.

To investigate whether *H. hepaticus* directly induces IL-10 in macrophages, we generated M-CSF differentiated bone marrow-derived macrophages (BMDMs) and subjected them to stimulation with whole bacteria (*Hh,* strain ATCC51449), a filtered supernatant of *H. hepaticus* culture medium (SN*Hh*) or the control culture medium (TSB). Measurement of cytokine gene transcription and protein expression after 3 hr stimulation showed that *Hh* induced high amounts of IL-10 and moderate IL-6 and TNFα (Figures 2A and S2A). Interestingly, SN*Hh* was sufficient to induce equivalent amounts of IL-10 in BMDMs, but a markedly diminished amount of IL-6 and TNFα compared to whole bacteria (Figures 2A and S2A). Treatment of SN*Hh* (SN*Hh*t) with DNase, RNase, and proteinase K followed by heat (2 hr, 95°C) had no effect on its capacity to induce IL-10 production in BMDMs, but significantly attenuated both IL-6 and TNFα production (Figures 2B, 2C, S2B, and S2C). This suggests that the IL-10-inducing factor produced by *H. hepaticus* is not a nucleic acid or a protein, and therefore likely a polysaccharide. Fractionation by size using Vivaspin concentrator columns showed that the ability to induce IL-10 in BMDMs was restricted to a high molecular weight component (SN*Hh*t > 30 kDa), indicating that the active molecule is a large polysaccharide (Figure 2B). Stimulation with SN*Hh*t and SN*Hh*t > 30 kDa resulted in significantly higher IL-10/IL-6 and IL-10/TNFα ratios compared to whole *H. hepaticus* and non-treated SN*Hh* (Figures 2D and S2C). Comparison with a known bacteria-derived immunostimulatory ligand, ultra-pure lipopolysaccharide from *Escherichia coli* (LPS UP), showed that the latter produced a more mixed cytokine response with large amounts of IL-10, IL-6, and TNFα, resulting in lower IL-10/IL-6 and IL-10/TNFα ratios compared to SN*Hh*t (Figures 2B–2D and S2C). SN*Hh*t induced IL-10 in a dose-dependent manner, but the IL-10/IL-6 and IL-10/TNFα ratios remained constant (Figure S2D). This suggests that the high ratios induced by SN*Hh*t are not a function of the stimulus concentration and reflect a qualitative response.

To further characterize the active molecule released into *H. hepaticus* culture supernatant that differentially stimulates IL-10 production, we oxidized SN*Hh*t using sodium metaperiodate (NaIO_4_) and dialyzed it against water to remove traces of reagent. This treatment cleaves sugars in the polysaccharide chains. Strikingly, after NaIO_4_ oxidation, SN*Hh*t lost its capacity to induce IL-10 (Figure 2E), supporting the idea that the active molecule in SN*Hh*t is a polysaccharide. This result was not a consequence of cellular toxicity of residual NaIO_4_ as simultaneous stimulation with both TSBt treated with NaIO_4_ and standard *E. coli* LPS (LPS ST) triggered IL-10 production by BMDMs (Figure 2E). To complement this approach, we subjected SN*Hh*t to incubation with different lectin-coated agarose beads. Lectins are carbohydrate-binding proteins with high specificity for mono- and oligosaccharides that also bind a diversity of complex polysaccharides. Our results showed that Concanavalin A from *Canavalia ensiformis* (ConA)-coated beads could deplete the IL-10-inducing factor from SN*Hh*t in a dose-dependent manner (Figure 2F), whereas other lectin-coated beads (lectins from *Arachis hypogea*, Peanut Agglutinin (PNA) or *Lens Culinaris* (LcH) had little effect on SN*Hh*t-induced IL-10 (Figure S2E). The lectins did not directly affect the capacity of BMDMs to produce IL-10, as shown by the simultaneous stimulation with both TSBt treated with ConA-coated beads and LPS ST (Figure 2F). Depletion by ConA suggests that SN*Hh*t is rich in α-mannose and α-glucose sugars. Finally, we used cold-ethanol precipitation to isolate the crude polysaccharide fraction (CP*Hh*t) from the treated supernatant (SN*Hh*t). CP*Hh*t recapitulated SN*Hh*t activity on BMDMs, with induction of high IL-10 but low IL-6 production (Figures 2G and S2B).

Collectively, these data indicate that *H. hepaticus* releases a large polysaccharide into the culture medium that induces macrophages to produce a selective response with large amounts of IL-10 and smaller amounts of pro-inflammatory cytokines such as IL-6 and TNFα.

### *H. hepaticus* Supernatant Is Sufficient to Induce IL-10 *In Vivo*

**Figure 3:**
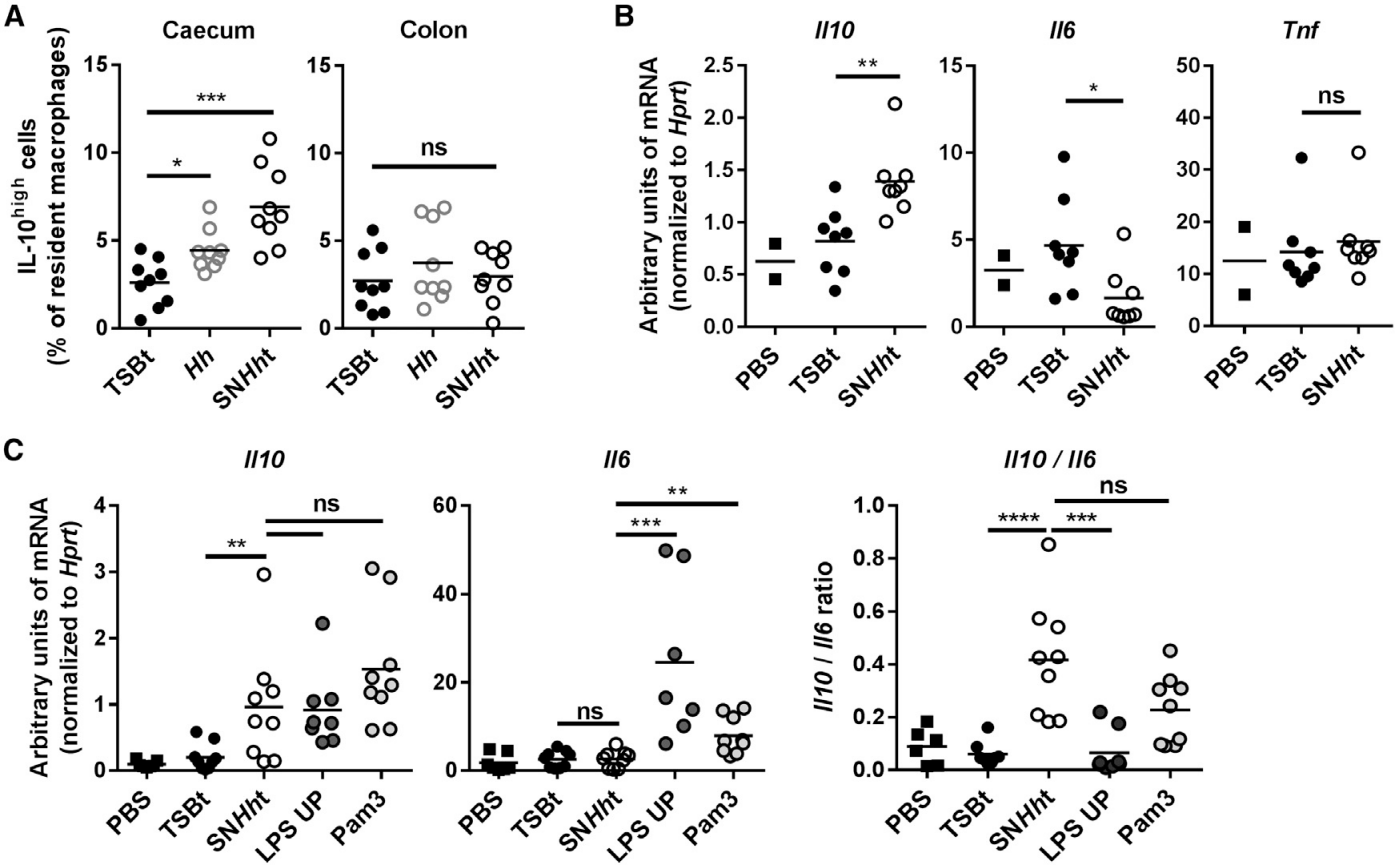
SN*Hh*t Is Sufficient to Induce IL-10 *In Vivo* (A) Frequency of IL-10^high^ cells among resident macrophages from caecum and colon LPL. SPF WT mice were infected with *Hh* or orally gavaged with TSBt or SN*Hh*t for 3 days. (B and C) *Il10*, *Il6*, and *Tnf* mRNA expression levels in the peritoneal cell fraction after 2 days (B) or *Il10* and *Il6* mRNA transcripts after 6 hr (C) challenge. Ligands (TSBt and SN*Hh*t, 200 μL; LPS UP and Pam3, 50 μg) were injected into the intraperitoneal cavity of SPF WT mice. Each symbol represents an individual mouse (two to three independent experiments). Pam3, Pam3CSK4. Mann-Whitney test, p < 0.05. See also Figure S3.

To assess the activity of *H. hepaticus* polysaccharide *in vivo*, we orally administered TSBt, SN*Hh*t, or live *H. hepaticus* to SPF mice daily for 2 days and analyzed the colon and caecum LPL by flow cytometry on day 3. Like *H. hepaticus* infection (Figure 1), SN*Hh*t treatment similarly increased the frequency of IL-10^high^ intestinal macrophages in the caecum, but not in the colon (Figures 3A, S3A, and S3B).

To test the activity of SN*Hh*t in a different immune compartment, we injected TSBt or SN*Hh*t intraperitoneally either daily for 2 days with analysis 48 hr after the first administration or as a single injection with analysis after 6 hr. In both conditions, SN*Hh*t induced a significant increase in *Il10* mRNA in total peritoneal cells compared to TSBt controls, with no change in *Il6* or *Tnf* (Figures 3B, 3C, and S3C), similar to the *in vitro* observations. Pam3CSK4 (Pam3) and LPS UP injections induced similar *Il10* mRNA but higher amounts of *Il6* (Figure 3C) and *Tnf* (non-significant; Figure S3C) in total peritoneal cells after 6 hr.

### SN*Hh*t Signals through TLR2 and MyD88 to Induce IL-10

**Figure 4:**
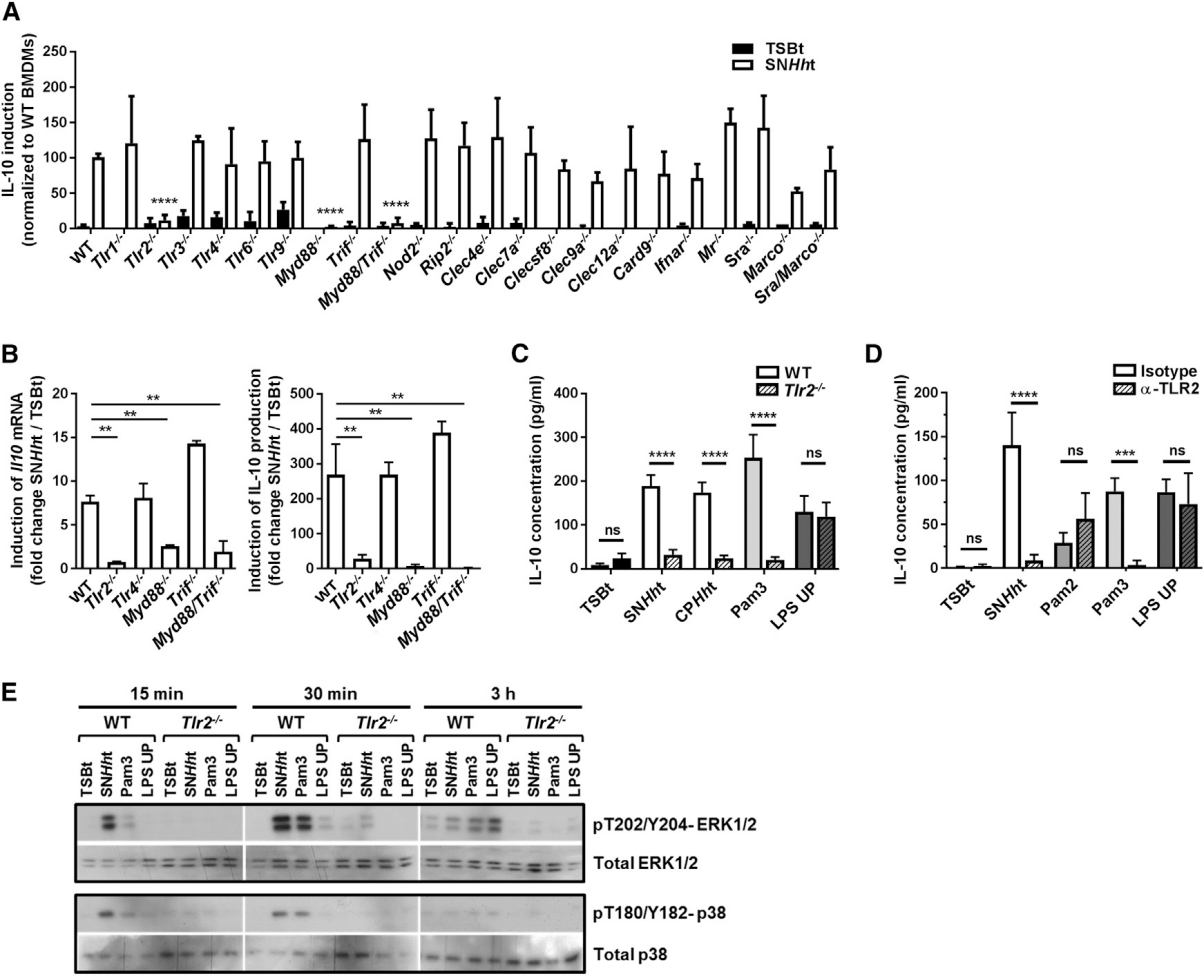
SN*Hh*t Signals through TLR2 and MyD88 to Induce IL-10 (A) Induction of IL-10 production in different knockout C57BL/6 BMDMs stimulated for 3 hr with SN*Hh*t normalized to WT BMDMs (three independent experiments). Two-way ANOVA and Bonferroni’s multiple comparisons test, p < 0.05. *Ifnar*, interferon-a/b receptor; *Mr*, mannose receptor; *Sra*, scavenger receptor A. (B) *Il10* mRNA and protein induction in different knockout BMDMs stimulated for 3 hr with SN*Hh*t (one of three independent experiments). One-way ANOVA and Tukey’s multiple comparisons test, p < 0.05. (C) Induction of IL-10 production in WT and *Tlr2*^−/−^ BMDMs stimulated for 3 hr with TSBt, SN*Hh*t, CP*Hh*t (SN*Hh*t crude polysaccharide fraction), LPS UP (TLR4 ligand), or Pam3 (TLR2/1) (one of three independent experiments). Two-way ANOVA and Sidak’s multiple comparisons test, p < 0.05. (D) Induction of IL-10 production in WT BMDMs pre-treated for 2 hr with a blocking antibody specifically inhibiting TLR2 signaling (monoclonal α-mTLR2-IgG) or its isotype control before stimulation for 3 hr with TSBt, SN*Hh*t, Pam2 (TLR2/6), Pam3, or LPS UP (three independent experiments). Two-way ANOVA and Sidak’s multiple comparisons test, p < 0.05. (E) Western blot showing the phosphorylation of ERK1/2 and p38 and the total ERK1/2 and p38 protein amounts in WT and *Tlr2*^−/−^ BMDMs after stimulation with TSBt, SN*Hh*t, Pam3, or LPS UP for 15 min, 30 min, and 3 hr. Mean ± SD. See also Figure S4.

To determine which sensing pathways were used by macrophages for IL-10 induction, we stimulated a panel of knockout BMDMs deficient in signaling proteins or PRRs—such as TLRs, NOD-like receptors (NLRs), and C-type lectin receptors (CLRs)—with SN*Hh*t or TSBt. In the response to bacterial components, TLR activation leads to recruitment of the adaptor molecules MyD88 (myeloid differentiation primary response gene 88) and/or TRIF (Toll-interleukin receptor domain containing adaptor-inducing interferon-γ), and switches on signaling networks that induce the production of various cytokines including IL-10, IL-6 and TNFα. Interestingly, *Myd88*^−/−^ and *Myd88*^−/−^*Trif*^−/−^, but not *Trif*^−/−^ BMDMs, lost their capacity to induce IL-10 at both transcriptional and protein levels in response to a 3 hr stimulation with SN*Hh*t (Figures 4A and 4B). Among the TLR-deficient cells tested (*Tlr1*^−/−^, *Tlr2*^−/−^, *Tlr3*^−/−^, *Tlr4*^−/−^, *Tlr6*^−/−^, and *Tlr9*^−/−^), only *Tlr2*^−/−^ BMDMs failed to produce IL-10 after stimulation with SN*Hh*t (Figures 4A and 4B), suggesting that SN*Hh*t signals through the TLR2/MyD88 pathway. As expected, the crude polysaccharide fraction CP*Hh*t and the canonical TLR2/1 ligand Pam3 were not able to induce IL-10 in *Tlr2*^−/−^ BMDMs, contrary to the TLR4 ligand LPS UP (Figure 4C). By contrast, BMDMs deficient for CLRs (MINCLE, CLEC4E; DECTIN-1, CLEC7A; MCL, CLECSF8; DNGR1, CLEC9A; MICL, CLEC12A), other receptors (IFNAR, interferon-α/β receptor; MR, Mannose Receptor; SRA, Scavenger Receptor A; MARCO; NOD2) or signaling proteins (RIP2; CARD9) were still able to produce IL-10 after stimulation with SN*Hh*t (Figure 4A).

As a complementary approach, a TLR2 blocking monoclonal antibody or an isotype control was added prior to BMDM stimulation with SN*Hh*t or different canonical TLR ligands. TLR2 blockade inhibited IL-10 induction by SN*Hh*t and Pam3 (TLR2/1 ligand) but not by Pam2CSK4 (Pam2, TLR2/6) or LPS UP (TLR4) (Figure 4D). Downstream of TLR2 and MyD88, the mitogen-activated protein kinase (MAPK) TAK1 acts as an activator of several kinases, including the MAPKs ERK1/2, p38, and c-*jun* N-terminal kinase (JNK), which converge on transcription factors such as NF-κB. Pharmacological blockade of these downstream kinases showed that IL-10 induction by SN*Hh*t involves TAK1, MEK1/2 (ERK pathway), and p38, with comparatively weak contributions by JNK and NF-κB (Figure S4A). Immunoblot analysis showed marked phosphorylation of ERK1/2 (pT202/Y204) and p38 (pT180/Y182) MAPKs in WT BMDMs stimulated with SN*Hh*t at early time points (15 and 30 min) compared to Pam3 and LPS UP, with no change in total protein amounts (Figure 4E). As expected, the phosphorylation pattern induced by SN*Hh*t and Pam3 was lost in *Tlr2*^−/−^ BMDMs (Figure 4E). In addition, as SN*Hh*t signals through TLR2, we compared its capacity to induce IL-10, IL-6, and TNFα in BMDMs to various canonical TLR2 and TLR4 ligands. Interestingly, SN*Hh*t induced higher IL-10/IL-6 and IL-10/TNFα ratios compared to Pam2 (TLR2/6 ligand), Pam3 (TLR2/1), FSL-1 (TLR2/6), zymosan from *Saccharomyces cerevisiae* (TLR2), lipomannan from *Mycobacterium smegmatis* (TLR2), LPS UP (TLR4), and LPS ST (TLR4 and TLR2) (Figure S4B).

These data indicate that TLR2/MyD88 is the pathway required for SN*Hh*t signaling, and furthermore that SN*Hh*t is a stronger driver of anti-inflammatory activity in macrophages than canonical TLR2 agonists.

### Differential Regulation of Transcription by SN*Hh*t and Pam3

**Figure 5:**
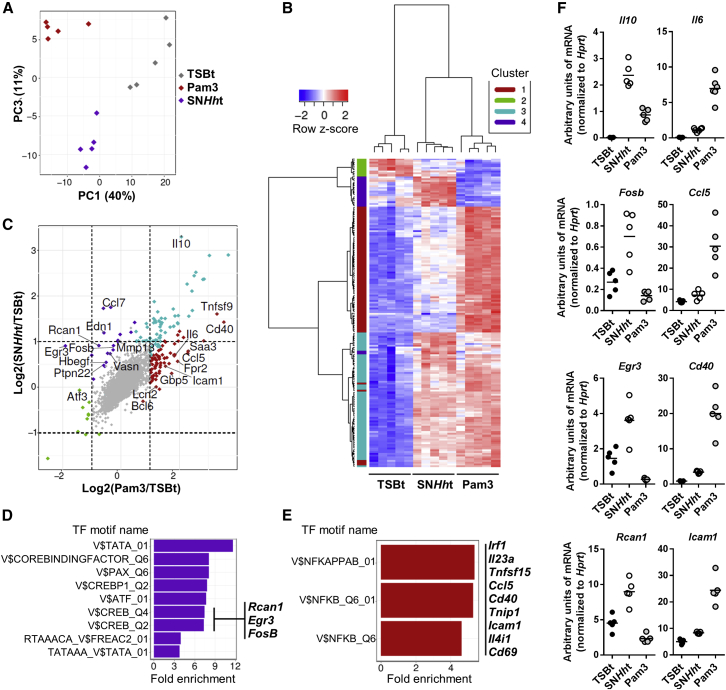
Differential Regulation of Transcription by SN*Hh*t and Pam3 (A–F) Microarray analysis performed on M-CSF BMDMs differentiated from 5 WT mice and stimulated for 3 hr. (A) Principle components analysis (PCA) of transcriptional profiles (empirical Bayes normalized expression values from LIMMA) across three conditions (TSBt, Pam3, and SN*Hh*t, n = 5/condition, 16,692 probes). (B) Hierarchical clustering heatmap (Manhattan distance with Ward clustering of empirical Bayes normalized expression values) of the union of differentially expressed probes between any condition-condition contrast (i.e., TSBt versus Pam3, TSBt versus SN*Hh*t, or Pam3 versus SN*Hh*t) (n = 172 probes, n = 146 genes). Probes were assigned to clusters using k-means clustering (k = 4) and cluster assignments are annotated as colors to the left of the heatmap. The number of probes assigned to each cluster were: cluster 1 = 75, cluster 2 = 10, cluster 3 = 68, and cluster 4 = 19. (C) Scatterplot displaying the relationship between log2 (fold changes) for each gene shown in (B) obtained when comparing either Pam3 (x axis) or SN*Hh*t (y axis) with the TSBt control condition. Colors represent the cluster assignments. (D and E) Transcription factor (TF) motif enrichment analysis among genes assigned to (D) cluster 4 (i.e., specifically induced by SN*Hh*t) and (D) cluster 1 (i.e., specifically induced by Pam3). Motif names correspond to Transfac motif identifiers derived from the Molecular Signatures Database (MSigDB). Motifs significantly overrepresented at an adjusted p < 0.05 (permutation test) are shown. (F) mRNA levels of CREB (*Il10*, *Fosb*, *Egr3*, *Rcan1*) and NF-κB (*Il6*, *Ccl5*, *Cd40*, *Icam1*) target genes were validated by qRT-PCR.

In order to assess whole genome differences in the macrophage transcriptional response to SN*Hh*t or to the canonical TLR2/1 agonist Pam3, we performed a microarray analysis of TSBt, SN*Hh*t, or Pam3-stimulated M-CSF differentiated BMDMs. Principle components analysis (PCA) demonstrated clear gene expression differences in macrophages stimulated by SN*Hh*t compared to Pam3 (Figure 5A). Hierarchical clustering analysis of differentially regulated genes identified four distinct clusters of co-regulated genes (Figure 5B). Each gene was assigned to a distinct cluster using k-means clustering (k = 4), representing genes that were (1) induced by Pam3 only (red cluster 1, n = 75); (2) repressed by both SN*Hh*t and Pam3 (green cluster 2, n = 10); (3) induced by both SN*Hh*t and Pam3 (blue cluster 3, n = 68); and (4) induced by SN*Hh*t only (purple cluster 4, n = 19). A large repertoire of pro-inflammatory genes was associated with the Pam3-specific transcriptional signature (Figure 5C). These included a number of genes associated with M1 pro-inflammatory macrophages (*Il6*, *Saa3*, *Ccl5*, *Lcn2*, and *Fpr2*) and others involved in the recruitment, activation, and proliferation of T cells (*Cd40* or *Tnfrsf5*, *Tnfrsf9*, *Icam1*) (Chen and Flies, 2013, Lebedeva et al., 2005, Liu et al., 2013, Zigmond et al., 2014). By contrast, SN*Hh*t specifically induced the transcription of a number of genes that are highly expressed in tissue resident or M2 macrophages (*Ccl7*, *Mmp13*, *Ptpn22*) (Chang et al., 2013, Shi and Pamer, 2011, Toriseva et al., 2012), transcription factors known to repress the NF-κB pathway (*Rcan1, Atf3*) and T cell activation (*Egr3*), and genes involved in tissue repair and wound healing (*Edn1*, *Mmp13*, *Hbegf*) (Figure 5C) (Cheluvappa et al., 2014, De Nardo et al., 2014, Junkins et al., 2013, Safford et al., 2005, Toriseva et al., 2012, Wei et al., 2015). Of note, we observed that although the anti-inflammatory cytokine IL-10 was induced by both SN*Hh*t and Pam3 (compared to TSBt), the strength of induction was greater for SN*Hh*t, consistent with a lower capacity to induce a pro-inflammatory response upon SN*Hh*t stimulation (Figure 5C).

We reasoned that the differential transcriptional programs activated in response to these two TLR2 agonists were driven by alternative transcription factor profiles. To test this, we performed enrichment analysis of transcription factor motif gene sets from the Molecular Signatures Database (MSigDB) among genes that were specifically induced by either SN*Hh*t (purple cluster 4) or Pam3 (red cluster 1). Specific targets induced by SN*Hh*t were enriched for genes (*Rcan1*, *Egr3*, *Fosb*) with predicted binding sites for multiple transcription factors that included two motifs for the cyclic AMP response-element binding protein (CREB) (Figure 5D). This is of interest as CREB is a key transcriptional regulator known to promote anti-inflammatory signaling and repair (Lawrence and Natoli, 2011, Wen et al., 2010). Therefore, CREB activation might play a role in the suppression of TLR2-induced pro-inflammatory responses by SN*Hh*t. In addition, the observation of a more pro-inflammatory transcriptional program in response to Pam3 was confirmed through the identification of a significant enrichment of genes with predicted NF-κB binding sites among genes that were specifically induced by Pam3 stimulation (Figure 5E). Differential induction of each gene of interest, including targets of CREB (*Il10*, *Fosb*, *Egr3*, *Rcan1*) and NF-κB (*Il6*, *Ccl5*, *Cd40*, *Icam1*), was validated by qRT-PCR (Figure 5F).

Taken together, these data suggest that TLR2 activation in BMDMs results in markedly different transcriptional programs depending on the agonist. The increased anti-inflammatory properties of SN*Hh*t are likely dependent on its ability to activate CREB and repress the pro-inflammatory cytokine response.

### SN*Hh*t Stimulates CREB Phosphorylation and Induction of an Anti-inflammatory and Repair Gene Signature in Macrophages

**Figure 6:**
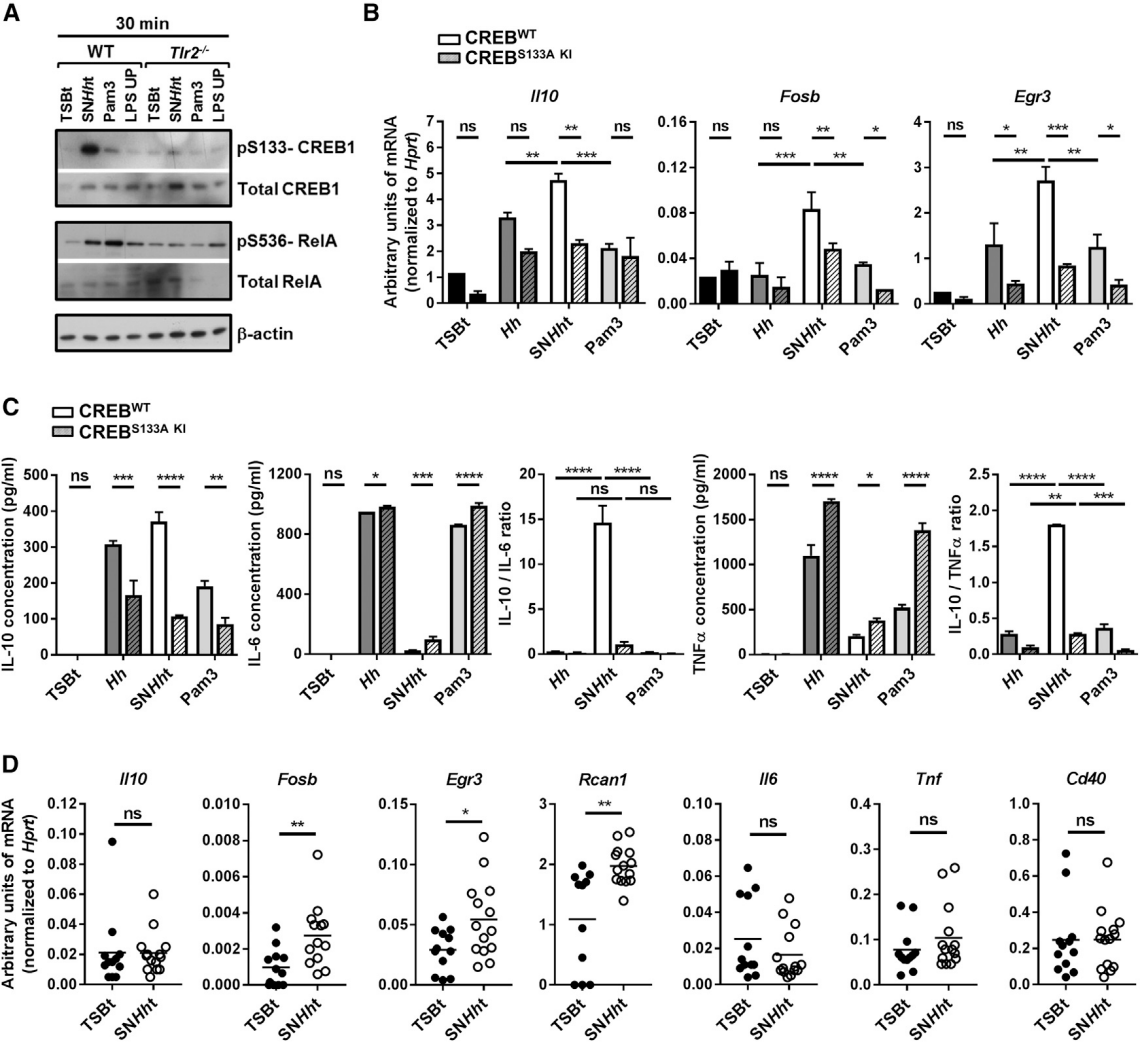
SN*Hh*t Stimulates CREB Phosphorylation and Induction of an Anti-inflammatory and Repair Gene Signature in Macrophages (A) Western blot showing the phosphorylation of CREB1 S133 and RelA S536 and the total CREB1 and RelA protein amounts in WT and *Tlr2*^−/−^ BMDMs after 30 min stimulation with TSBt, SN*Hh*t, Pam3, or LPS UP. β-actin used as a loading control. (B) mRNA levels of *Il10*, *Fosb*, and *Egr3* genes in BMDMs from conditional *vav-cre* CREB^WT^ and CREB^S133A KI^ mice after 1 hr stimulation with TSBt, *Hh*, SN*Hh*t, or Pam3. (C) Induction of IL-10, IL-6, and TNFα; IL-10/IL-6; and IL-10/TNFα protein ratios in BMDMs from conditional *vav-cre* CREB^WT^ and CREB^S133A KI^ mice after 10 hr stimulation with TSBt, *Hh*, SN*Hh*t, or Pam3. One of three independent experiments. Two-way ANOVA and Sidak’s and/or Tukey’s multiple comparisons tests, p < 0.05. (D) mRNA levels of *Il10*, *Fosb*, *Egr3*, *Rcan1*, *Il6*, *Tnf*, and *Cd40* genes in caecum total tissue from WT mice gavaged with TSBt or SN*Hh*t for 2 days. Each symbol represents an individual mouse (two independent experiments). Mann-Whitney test, p < 0.05. Mean ± SD. See also Figure S5.

Immunoblot analysis of pS133-CREB1 and pS536-RelA in BMDMs revealed differential induction of CREB and NF-κB pathways after 30 min stimulation with SN*Hh*t, Pam3, or LPS UP (Figure 6A). Consistent with the microarray data, SN*Hh*t induced stronger CREB but weaker RelA phosphorylation compared to Pam3, and this pattern was lost in *Tlr2*^−/−^ cells (Figure 6A). To test the functional role of CREB^S133^ phosphorylation in the induction of the CREB target genes identified in Figure 5, we differentiated BMDMs from conditional CREB^S133A KI^ (Serin replaced by Alanin in position 133 of the CREB protein) and CREB^WT^ mice. These mice were both generated using a minigene strategy (Wingate et al., 2009) with a CRE recombinase under the *vav* promoter, expressed throughout the hematopoietic compartment. CREB could not be phosphorylated at Ser133 in conditional *Vav*-cre CREB^S133A KI^ BMDMs after stimulation with SN*Hh*t (Figure S5A). Stimulation of conditional *Vav*-cre CREB^S133A KI^ and CREB^WT^ BMDMs with SN*Hh*t for 1 hr showed significant induction of the CREB target genes *Il10*, *Fosb*, and *Egr3* relative to stimulation with whole *H. hepaticus* or Pam3 (Figure 6B). The induction of *Il10*, *Fosb*, and *Egr3* was partially dependent on CREB phosphorylation, whereas *Il6*, *Tnf*, or *Cd40* induction was unaffected (Figures 6B and S5B). Antibody blockade of the IL-10R showed that induction of CREB target genes, specifically *Fosb*, *Egr3*, and *Rcan1*, was not due to an IL-10 autocrine pathway (Figure S5C). Interestingly, after 10 hr stimulation, CREB^S133A KI^ BMDMs produced less IL-10 but more IL-6 and TNFα in response to SN*Hh*t compared to CREB^WT^ BMDMs (Figure 6C). This led to strongly reduced IL-10/IL-6 and IL-10/TNFα ratios, reaching levels similar to those observed with whole *H. hepaticus* or Pam3 (Figure 6C). Importantly, the induction of anti-inflammatory CREB targets (*Fosb*, *Egr3*, *Rcan1*) but not pro-inflammatory NF-κB targets (*Il6*, *Tnf*, *Cd40*) was also observed in total caecum tissue from mice gavaged for 2 days with SN*Hh*t (Figure 6D). These data indicate that CREB phosphorylation promotes the anti-inflammatory properties of SN*Hh*t and that this pathway is operational in the intestine.

### MSK1/2 is Essential for the Anti-inflammatory Properties of SN*Hh*t

**Figure 7:**
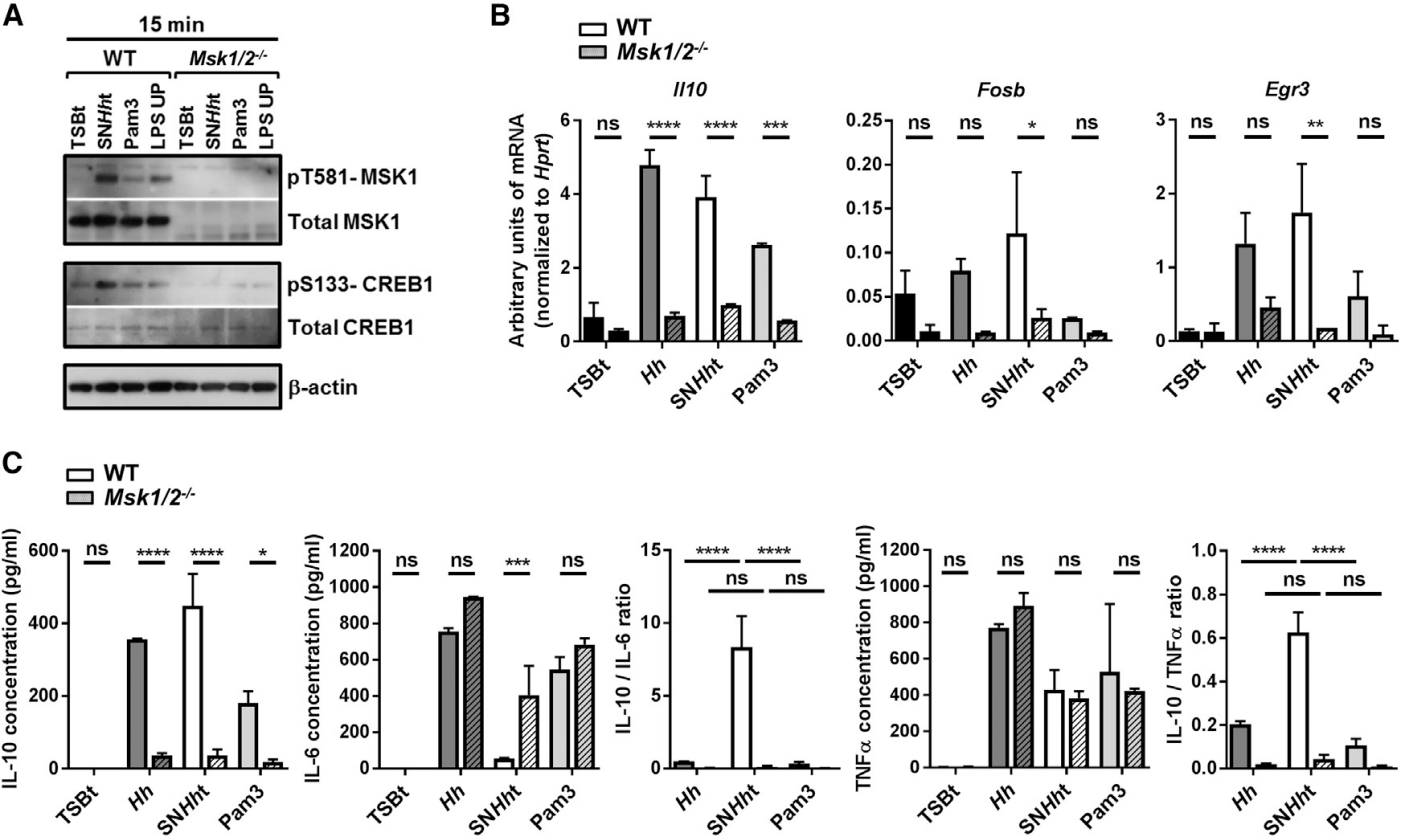
MSK1/2 Is Essential to the Anti-inflammatory Properties of SN*Hh*t (A) Western blot showing the phosphorylation of CREB1 S133 and MSK1 T581 and the total CREB1 and MSK1 protein amounts in WT and *Msk1/2*^−/−^ BMDMs after 30 min stimulation with TSBt, SN*Hh*t, Pam3, or LPS UP. (B) mRNA levels of *Il10*, *Fosb*, and *Egr3* genes in BMDMs from WT or *Msk1/2*^−/−^ mice after 1 hr stimulation. (C) Induction of IL-10, IL-6, and TNFα; IL-10/IL-6; and IL-10/TNFα protein ratios in BMDMs from WT or *Msk1/2*^−/−^ mice after 10 hr stimulation. One of three independent experiments. Two-way ANOVA and Sidak’s and/or Tukey’s multiple comparisons tests, p < 0.05. Mean ± SD. See also Figure S6.

The mitogen and stress-activated protein kinase (MSK) isoforms, MSK1 and MSK2, are activated downstream of p38 and ERK1/2 *in vivo* and phosphorylate CREB, triggering the rapid transcription of CREB target genes (Ananieva et al., 2008). We stimulated BMDMs differentiated from WT and *Msk1/2*^−/−^ mice with SN*Hh*t. Immunoblot analysis of pS133-CREB1 and pT581-MSK1 revealed that SN*Hh*t phosphorylates MSK1 and that SN*Hh*t-mediated CREB phosphorylation requires MSK1/2 (Figure 7A). The stimulation of WT and *Msk1/2*^−/−^ BMDMs with SN*Hh*t for 1 hr confirmed that the induction of CREB target genes (*Il10*, *Fosb*, and *Egr3*) is highly dependent on MSK1/2, contrary to the induction of *Il6*, *Tnf*, or *Cd40* (Figures 7B and S6). As expected, *Msk1/2*^−/−^ BMDMs produced less IL-10 but more IL-6 and TNFα in response to 10 hr stimulation with SN*Hh*t. The *Msk1/2* deletion led to a strong reduction of both IL-10/IL-6 and IL-10/TNFα ratios, such that they were indistinguishable from the ratios induced by whole *H. hepaticus* or Pam3 (Figure 7C). Together, these data indicate that both MSK1/2 and CREB are required for the anti-inflammatory properties of SN*Hh*t.

## Discussion

The intestine is home to billions of bacteria, and this complex ecosystem plays an important role in health and disease. It is well appreciated that microbial induction of IL-10 in the intestine is a non-redundant feature of tolerance to the microbiota, but the molecular dialog between host and microbe that determines tolerance and immunity remains elusive. Here, we show that *H. hepaticus* strain ATCC51449 produces a large polysaccharide that shapes the macrophage response. This polysaccharide triggers a high IL-10/IL-6 ratio compared to canonical TLR2 agonists and selectively activates the transcription factor CREB that, in turn, induces a panel of anti-inflammatory and repair mediators in the intestine.

*H. hepaticus* can be considered as a pathosymbiont based on its ability to live in harmony with its murine host in the presence of an IL-10 response, and its potential to drive IL-23-driven intestinal inflammation in IL-10 deficiency. The bacterial-sensing pathways that mediate these distinct responses are poorly understood. Our current understanding derives from *in vitro* studies in BMDMs showing that the activation of ERK inhibits the induction of IL-12 p40 by *H. hepaticus* (Tomczak et al., 2006). Moreover, epithelial cell lines transfected with human TLR2 could recognize the whole bacteria (Mandell et al., 2004). A key role for the adaptor MyD88 has been shown *in vivo* in the inflammatory response to *H. hepaticus* in IL-10 deficiency, but the upstream sensors were not identified (Asquith et al., 2010, Boulard et al., 2010). Our results show that *H. hepaticus* produces a polysaccharide that induces a specific CREB-dependent anti-inflammatory and repair gene signature in macrophages via the TLR2 pathway. CREB targets include *Il10* as well as transcription factors that downregulate NF-κB and T cell activation, and genes involved in tissue repair. The transcription factor CREB is known to limit pro-inflammatory signaling downstream of TLRs and stimulate *Il-10* transcription (Cohen, 2014, Wen et al., 2010). Previous studies have shown TLR-mediated activation of the CREB pathway. For example, LPS stimulation of BMDMs induced anti-inflammatory genes in an MSK/CREB-dependent manner (Ananieva et al., 2008, Darragh et al., 2010). Similarly, the TLR2/DECTIN-1 ligand zymosan and TLR2/4-signaling extracts from *Schistosoma mansoni* cercariae were able to activate CREB in BMDMs and induce various CREB target genes that are associated with a “regulatory” macrophage phenotype or cell metabolism, respectively (Elcombe et al., 2013, Sanin et al., 2015). Here, we identified a bacterial factor able to activate a CREB-dependent anti-inflammatory program both *in vitro* and *in vivo*, suggesting that the CREB pathway might play a crucial role in host-microbiota mutualism.

IL-10 itself inhibits the production of pro-inflammatory cytokines in macrophages through Jak–STAT3 activation (Moore et al., 2001, Murray, 2006); however, we found that the early induction of CREB targets by SN*Hh*t is independent of IL-10R signaling but rather relies on MSK1/2 kinase activity. Activated by p38 and ERK1/2, MSK1/2 phosphorylates multiple substrates, including CREB, ATF1, and Histone H3, and predominantly has anti-inflammatory roles (Reyskens and Arthur, 2016). Here, the deletion of MSK1/2 has a larger effect on SN*Hh*t-induced anti-inflammatory ratios compared to the loss of CREB^S133^ phosphorylation. This suggests that MSK1/2 activates both CREB-dependent and independent anti-inflammatory pathways in response to SN*Hh*t, potentially involving Histone H3-mediated epigenetic changes. As SN*Hh*t also induces this CREB/MSK-dependent immunomodulatory program in intestinal tissue *in vivo*, it is likely that it contributes to mutualistic relationships in the *H. hepaticus*-infected gut. Further studies are required to test this hypothesis.

IL-10 was previously shown to be critical for macrophage conditioning within the intestinal tissue, but IL-10 production by macrophages per se is not sufficient to explain their homeostatic activity (Shouval et al., 2014, Zigmond et al., 2014). The ability of SN*Hh*t to induce a CREB-driven anti-inflammatory response that is IL-10 independent may reveal additional checkpoints that control the macrophage inflammatory response in the intestine. *H. hepaticus* also promotes IL-10-producing regulatory T cells (Kullberg et al., 2002) raising the possibility that SN*Hh*t-conditioned macrophages promote this response.

By contrast with SN*Hh*t, the canonical TLR2 ligand Pam3 induced a broad NF-κB-dependent pro-inflammatory response in BMDMs. Microbe-induced TLR2 signaling promotes both pro- and anti-inflammatory responses (Nemati et al., 2017, Oliveira-Nascimento et al., 2012). However, it is not known whether this is a consequence of the structural features of particular ligands or whether it reflects the activities of additional sensors and receptors. CLRs, such as DECTIN-1 and DC-SIGN, have been shown to collaborate with TLRs to select specific responses to infectious agents (Ferwerda et al., 2008, Gantner et al., 2003). Here, we have ruled out the involvement of several CLRs and the common downstream CLR-signaling protein CARD9 in IL-10 induction by SN*Hh*t. Progress in this area is hampered by the often complex bacterial cell wall structures. Despite considerable effort, we were not able to analyze the structure and composition of the polysaccharide produced by *H. hepaticus*. The genome of *H. hepaticus* ATCC51449 does not contain a canonical polysaccharide biosynthesis operon, but many polysaccharide glycosyltransferase and LPS synthesis genes (Lombard et al., 2014). The LPS from *H. hepaticus* CCUG33637 was described as a low molecular weight molecule (Hynes et al., 2004), making a similar molecule an unlikely candidate. However, we cannot rule out that the high molecular weight polysaccharide produced by *H. hepaticus* ATCC51449 also contains lipid moieties. Additional analyses are required to decipher the structure and the conservation of this molecule across species. One approach is to sequence different strains of *H. hepaticus* to determine whether this activity is unique to a particular strain or a more general feature. In preliminary studies, we found that *H. hepaticus* strain ATCC51448 did not possess the same immunomodulatory activity as strain ATCC51449 (data not shown). Comparative analyses of the sequences of these two stains could help identify gene candidates associated with polysaccharide biosynthesis. However, these studies are in their infancy as only strain ATCC51449 has been fully sequenced (Suerbaum et al., 2003).

The host response to *H. hepaticus* shares some features with that elicited by *Bacteroides fragilis*, a member of the human gut microbiota that can induce abscesses and bacteraemia (Wexler, 2007). Challenge of *II-10^−/−^* mice with *B. fragilis* also leads to higher inflammation and mortality compared to WT mice, emphasizing the important role of IL-10 in host-microbe interactions (Cohen-Poradosu et al., 2011). Interestingly, *B. fragilis* polysaccharide A (PSA, strain NCTC 9343) was able to limit colitis in mice (Mazmanian et al., 2008). PSA activates TLR2 on plasmacytoid dendritic cells and regulatory T cells to induce immune regulatory functions, including IL-10 production and suppression of Th17 responses (Dasgupta et al., 2014, Round et al., 2011). PSA is a capsular zwitterionic polysaccharide (ZPS). ZPSs contain positive and negative repeating charges that are crucial to their immunomodulatory activity (Surana and Kasper, 2012). Recently, Lozupone et al. performed a genomic screen for human gut bacteria encoding ZPSs but did not identify *Helicobacter* species (Neff et al., 2016). Furthermore, in the genome of *H. hepaticus* strain ATCC51449, we did not find any significant BLAST hits to ZPS biosynthesis genes from *B. fragilis*, suggesting that these two bacteria produce different types of polysaccharide or employ different mechanisms of production. These results, together with our own, illustrate that the host utilizes TLR pathways not only to initiate host defense, but to bolster immune regulatory pathways that promote intestinal homeostasis (Rakoff-Nahoum et al., 2004).

In summary, our studies show that *H. hepaticus* produces an immunomodulatory polysaccharide that conditions the macrophage response via a TLR2/CREB-driven anti-inflammatory pathway. A better understanding of the molecular crosstalk between host cells and commensal bacteria is of major importance to decipher mutualistic mechanisms. Enhancement of these could help maintain homeostasis following disruptive challenges such as antibiotic treatment, enteric infection, stress, and food allergy, as well as aid restoration of a balanced dialog in the face of chronic inflammation. Identification of immunomodulatory ligands produced by commensals will require an integrated approach combining immunology, microbiology, bioinformatics, and biochemistry. This field of investigation is challenging but highly valuable as it could ultimately provide new preventive and therapeutic strategies for infectious and inflammatory diseases.

## STAR★Methods

### Key Resources Table

**Table:** 

REAGENT or RESOURCE	SOURCE	IDENTIFIER
**Antibodies**		

Anti-IL-10 APC antibody (clone JES5-16E3)	eBioscience	clone JES5-16E3, RRID: AB_469502
Mouse IgG2k isotype control (Rat, clone A95-1)	BD Pharmingen	clone A95-1, Cat# 556924
Anti-mTLR2-IgG (clone C9A12) monoclonal antibody	InvivoGen	clone C9A12, Cat# mabg-mtlr2
Mouse IgG2A isotype control (clone 20102)	Biotechne (R&D systems)	clone 20102, MAB003
CD11b (clone M1/70)	BioLegend, London, UK	clone M1/70 RRID: AB_312791
CD11c (clone N418)	BioLegend, London, UK	clone N418 RRID: AB_313771
MHCII (clone M5/114.15.2)	BioLegend, London, UK	clone M5/114.15.2, RRID: AB_313321
CD64 (clone X54-5/7.1)	BioLegend, London, UK	clone X54-5/7.1 RRID:AB_10612740
CD45 (clone 30-F11)	BD Biosciences, Oxford, UK	clone 30-F11
Total ERK1/2	Cell Signaling Technology	#4695
pT202/Y204-ERK1/2 (clone D13.14.4E)	Cell Signaling Technology	clone D13.14.4E, #4370
Total p38	Cell Signaling Technology	#9212
pT180/Y182-p38 (clone 3D7)	Cell Signaling Technology	clone 3D7, #9215
pS133-CREB1 (clone 87G3)	Cell Signaling Technology	clone 87G3, #9198
pT581-MSK1	Cell Signaling Technology	#9595
Total CREB1 (clone E306)	Abcam	clone E306, #ab32515
Total MSK1 (#AF2518)	R&D systems	Accession #O75582

**Bacterial and Virus Strains**		

*Helicobacter hepaticus* strain ATCC51449	ATCC	ATCC51449
*Helicobacter hepaticus* strain ATCC51448	ATCC	ATCC51448

**Biological Samples**		

Pam2CSK4	InvivoGen	Cat# tlrl-pm2s-1
Pam3CSK4	InvivoGen	Cat# tlrl-pms
FSL-1	InvivoGen	Cat# tlrl-fsl
Zymosan, cell wall from *Saccharomyces cerevisiae*	InvivoGen	Cat# tlrl-zyn
LM-MS, Lipomannan from *Mycobacterium smegmatis*	InvivoGen	Cat# tlrl-lmms1
LPS ultrapure from *E. coli* O111:B4	InvivoGen	Cat# tlrl-3pelps
LPS standard from *E. coli* O55:B5	InvivoGen	Cat# tlrl-b5lps

**Chemicals, Peptides, and Recombinant Proteins**		

Sodium metaperiodate	Alfa Aesar, Heysham, UK	CAS# 7790-28-5
Concanavalin A from Canavalia ensiformis (Jack bean), agarose conjugate	Sigma	L7555
Lectin from Arachis hypogaea (peanut) *agarose conjugate*	Sigma	L2507
Lectin from Lens culinaris (lentil), agarose conjugate	Sigma	L4018
U0126	Merck Chemicals	662005-1MG
*p38 MAP Kinase Inhibitor VIII, EO 1428*	Merck Chemicals	506163-5MG
NF-kB Activation Inhibitor II, JSH-23	Merck Chemicals	481408-5MG
*(5Z)-7-Oxozeaenol*	Calbiochem	499610
sp600125	Sigma	S5567

**Critical Commercial Assays**		

DuoSet ELISA kits	R&D Systems Europe – Biotechne	N/A
High-Capacity cDNA Reverse Transcriptase	Applied Biosystems, Life Technologies, Paisley, UK	N/A
PrecisionFAST mastermix	Primerdesign	N/A
TaqMan gene expression assays	Life Technologies	N/A
RNeasy Mini kit (for tissue and microarray)	QIAGEN, Manchester, UK	N/A
Quick-RNA MiniPrep kit (for primary cell cultures)	Zymo Research, Cambridge Bioscience, UK	N/A
Illumina MouseWG-6-V2 microarrays	Illumina	N/A

**Deposited Data**		

Microarray data	ArrayExpress	E-MTAB-5780

**Experimental Models: Organisms/Strains**		

*Mus musculus*, NCBI Taxonomy ID:10090, C57BL/6	Charles River	RRID:IMSR_JAX:000664

**Oligonucleotides**		

*Hprt*	TaqMan Gene Expression Assays for mouse, Life Technologies	Mm01545399_m1
*Il10*	TaqMan Gene Expression Assays for mouse, Life Technologies	Mm00439614_m1
*Il6*	TaqMan Gene Expression Assays for mouse, Life Technologies	Mm00446190_m1
*Tnf*	TaqMan Gene Expression Assays for mouse, Life Technologies	Mm00443258_m1
*Fosb*	TaqMan Gene Expression Assays for mouse, Life Technologies	Mm00500401_m1
*Egr3*	TaqMan Gene Expression Assays for mouse, Life Technologies	Mm00516979_m1
*Rcan1*	TaqMan Gene Expression Assays for mouse, Life Technologies	Mm01213406_m1
*Cd40*	TaqMan Gene Expression Assays for mouse, Life Technologies	Mm00441891_m1
*Icam1*	TaqMan Gene Expression Assays for mouse, Life Technologies	Mm00516023_m1
*Ccl5*	TaqMan Gene Expression Assays for mouse, Life Technologies	Mm01302427_m1

**Software and Algorithms**		

FlowJo software	Tree Star, Ashland, OR	N/A
GraphPad Prism 7.02	GraphPad Software, CA	N/A
Lumi	R/Bioconductor	N/A
LIMMA	R/Bioconductor	N/A
Molecular Signatures Database (MSigDB, C3, motif gene sets)	http://software.broadinstitute.org/gsea/msigdb/collections.jsp	N/A
Transfac identifier	http://www.gene-regulation.com/pub/databases.html	N/A
runGO.py from the Computational Genomics Analysis Toolkit (CGAT)	https://github.com/CGATOxford/cgat	N/A

**Other**		

Fortessa	BD Biosciences	N/A
ViiA 7 Real-Time PCR System	Life Technologies	N/A

### Contact for Reagent and Resource Sharing

Further information and requests for resources and reagents should be directed to and will be fulfilled by the Lead Contact, Fiona Powrie (fiona.powrie@kennedy.ox.ac.uk).

### Experimental Model Details

#### Mice

Wild-type C57BL/6 mice were bred and maintained under specific pathogen-free (SPF) conditions in accredited animal facilities at the University of Oxford. Experiments were conducted in accordance with the UK Scientific Procedures Act (1986) under a Project License (PPL) authorized by the UK Home Office. Mice were routinely screened and negative for *Helicobacter* spp. and other known intestinal pathogens and were over 6 week-old when experiments were started. Both males and females were used. Co-housed littermates of same sex were randomly assigned to experimental groups with the same number of males and females in the different experimental groups. Experimental groups were then kept in separated cages during treatment with TSB/SNHht or infection with *H. hepaticus*. *H. hepaticus* infection was performed by oral administration. Colonization with live *H. hepaticus* was controlled by qRT-PCR performed on total tissue mRNA using probes for the gene *cdtB*. 250 μL of 1mg/ml brefeldin A (from Penicillium Breffeldianu, Sigma-Aldrich Co) was injected intra-peritoneally 12 h before harvest for FACS staining experiments.

#### Bacterial Culture

The Gram-negative mouse pathobiont *Helicobacter hepaticus* strain ATCC51449 was grown in tryptone soya broth (TSB, Fisher) supplemented with 10% Fetal Calf Serum (FCS) and Skirrow Campylobacter supplements (Oxoid) in microaerophilic conditions (1%–3% oxygen). After 24 h at 37°C under agitation, the culture broth was centrifuged at 4500 rpm for 50 min and filtrated through a 0.2 mm filter. The supernatants were collected and stored at −20°C.

#### Culture of Mouse Bone-Marrow Derived Macrophages

Bone-marrow cells from SPF WT or knockout C57BL/6 mice (any sex) were cultured for 8 days in complete RPMI with 50 ng/ml murine M-CSF (Peprotech) and seeded at 100,000 cells on 96-well tissue culture plates overnight before stimulation. After differentiation with M-CSF, 90%–95% of bone-marrow derives macrophages (BMDMs) expressed CD11b and CD64, 15% MHCII and 5% CD11c by flow cytometry. Bone-marrow from knock-out and knock-in mice was provided by the collaborators listed in the Acknowledgments section.

### Methods Details

#### Isolation of Leukocytes from the Lamina Propria

For lamina propria leukocyte (LPL) isolation, the colon and the caecum of C57BL/6 mice were opened longitudinally, washed, and cut into pieces. Pieces were washed twice in RPMI-1640 medium supplemented with 5% fetal bovine serum (FBS) and 5mM EDTA at 37°C with shaking to remove epithelial cells. Tissue was then digested at 37°C for 1 h in a shaking incubator with 1 mg/ml type VIII collagenase (Sigma-Aldrich, Gillingham, UK) and 40 μg/ml DNase I in RPMI supplemented with 5% FBS. Cells were then layered on a 40/80% Percoll gradient, centrifuged, and the interface was recovered.

#### Flow Cytometry and Intracellular Staining

For surface staining, cells were first incubated with Fc block (anti-CD16/CD32, eBioscience) to minimize nonspecific antibody binding, and then stained for 20 min in phosphate-buffered saline / 0.1% bovine serum albumin / 5 mM EDTA buffer with a fixable viability dye and a combination of the following antibodies: CD11b (M1/70), CD11c (N418), MHCII (M5/114.15.2), CD64 (X54-5/7.1) (all from BioLegend, London, UK) and CD45 (30-F11) (from BD Biosciences, Oxford, UK). Following surface staining, cells were fixed with PFA 2%, permeabilized with saponin 0.05% and incubated with an anti-IL-10 APC antibody (clone JES5-16E3, eBioscience) at dilution 1/50 for 20 min. All cells were analyzed on a Fortessa (BD Biosciences) and analysis was performed using FlowJo software (Tree Star, Ashland, OR).

#### Treatment of *H. hepaticus* Culture Supernatant

SN*Hh*t was obtained by treating the filtered culture supernatant with 50 μg/ml DNase I (Roche) and RNase A (Roche) for 2 hours at 37°C, then with 40 μg/ml proteinase K (Sigma) overnight at 56°C, and finally with heat for 2 h in a water bath at 95°C. Protein and nucleic acid digestions were controlled using Sodium dodecyl sulfate polyacrylamide gel electrophoresis (SDS-PAGE) and agarose gels, respectively. To obtain SN*Hh*t > 30kDa and SN*Hh*t < 30kDa fractions, SN*Hh*t was concentrated on Vivaspin 20 MWCO 30 000 columns (GE healthcare Biosciences) for 30 min at 3000 rpm. Sodium metaperiodate oxidation was performed at 1 mM NaIO_4_ 98% (Alfa Aesar, Heysham, UK) in 0.1 M sodium acetate pH 5.5 for 6 h at RT in foil, followed by dialysis against distilled water for 2 days at 4°C to remove NaIO_4_ traces using Pur-A-Lyzer™ Maxi 3500 tubes (Sigma). Lectin agarose conjugates (Concanavalin A from *Canavalia ensiformis* (ConA), lectins from *Arachis hypogaea* (peanut - PNA) and *Lens culinaris* (lentil - LcH) (Sigma) were added to SN*Hh*t or TSBt to deplete the polysaccharide and incubated for 5 h on a wheel at RT, followed by 30 min rest and 10 min centrifugation at 13 000 rpm.

#### Ethanol Precipitation of the Crude Polysaccharide

CP*Hh*t was precipitated from SN*Hh*t by the addition of 4 volumes of ice cold ethanol and incubated at −20°C overnight. Crude polysaccharide fractions from TSBt (CPTSBt) were extracted using the same method as a control. Precipitated polysaccharides were recovered by centrifugation at 5000 rpm for 10 min, the pelleted precipitate was re-suspended in water and dialysed against 5 L of water for 48 h with two water changes to remove residual ethanol before being dried in a freeze dryer.

#### Stimulation of BMDMs

BMDM cultures were infected with 0.5 OD *H. hepaticus* in RPMI (from frozen stock of live culture, Multiplicity Of Infection = 10), *H. hepaticus* culture supernatant, TSBt control medium or different TLR ligands (Pam2CSK4 (85 ng/ml), Pam3CSK4 (75 ng/ml), FSL-1 (100 ng/ml), Zymosan, cell wall from *Saccharomyces cerevisiae* (100 ng/ml); LM-MS, Lipomannan from *Mycobacterium smegmatis* (1 ng/ml), LPS ultrapure from *E. coli* O111:B4 (20 ng/ml), LPS standard from *E. coli* O55:B5 (1 ng/ml) - all from InvivoGen) for 3 h at 37°C in a humidified incubator with 5% CO2. Blocking antibodies specifically inhibiting TLR2 signaling (Anti-mTLR2-IgG (C9A12) monoclonal antibody, InvivoGen) or a mouse IgG2A isotype control (Biotechne) were added at 0.3 μg/ml 1 h prior stimulation. Kinase inhibitors were used at 10 μM (UO126 (MEK1/2), JSH-23 (NFkb), Merck Chemicals; Sp600125 (JNK), Sigma), 5 μM (EO 1428 (p38), Merck Chemicals) or 1 μM (5z-7-oxozeanol (Tak1), Calbiochem) and added 30 min prior stimulation. Stimulation assays were performed in duplicates and repeated three or more times.

#### ELISA Assay

For measurement of secreted cytokines using Mouse IL-10, IL-6 and TNF-α DuoSet ELISA kits (R&D Systems Europe – Biotechne), culture supernatants were collected and stored at −20°C.

#### RNA Extraction

BMDMs or lamina propria cells were lysed in RLT buffer (QIAGEN, Manchester, UK) or Zymo Lysis Buffer (Zymo Research, Cambridge Bioscience, UK) and stored at −80°C. RNA was isolated from snap-frozen samples using the RNeasy Mini kit (QIAGEN, Manchester, UK) or the Quick-RNA MiniPrep kit (Zymo Research, Cambridge Bioscience, UK) according to the manufacturer’s instructions, including an on-column DNase I digestion step. For microarray analysis on BMDMs, the RNeasy Mini kit (QIAGEN, Manchester, UK) was used.

#### Quantitative RT-PCR

Complementary DNA synthesis was performed using High-Capacity cDNA Reverse Transcriptase (Applied Biosystems, Life Technologies, Paisley, UK). Quantitative PCR reactions for the candidate genes were performed using PrecisionFAST mastermix (Primerdesign) and TaqMan gene expression assays (Life Technologies). Complementary DNA samples were analyzed in duplicate using ViiA 7 Real-Time PCR System (Life Technologies), and gene expression levels for each sample were normalized to HPRT. Mean relative gene expression was determined, and the differences were calculated using the 2ΔC(t) method. Primer pairs and probes were as follows: TaqMan Gene Expression Assays for mouse *Hprt* (Mm01545399_m1), *Il10* (Mm00439614_m1), *Il6* (Mm00446190_m1), *Tnf* (Mm00443258_m1), *Fosb* (Mm00500401_m1), *Egr3* (Mm00516979_m1), *Rcan1* (Mm01213406_m1), *Cd40* (Mm00441891_m1), *Icam1* (Mm00516023_m1) and *Ccl5* (Mm01302427_m1).

#### Immunoblot Analysis

Cells were washed with PBS and lysed using Laemmli sample loading buffer (2% SDS, 10% glycerol, 50 mM DTT, 0.002% bromphenol blue and 62.5 mM Tris HCl, pH 6.8). Equal amounts of proteins were resolved by SDS–PAGE using NuPAGE Novex Bis-Tris Gels (Thermo Fisher Scientific) and analyzed with antibodies against total ERK1/2, pT202/Y204-ERK1/2 (D13.14.4E), total p38, pT180/Y182-p38 (3D7), pS133-CREB1 (87G3), pT581-MSK1 (all from Cell Signaling Technology), total CREB1 (E306, ab32515, Abcam) and total MSK1 (R&D systems) followed by detection with horseradish peroxidase (HRP)–conjugated secondary antibodies and the chemiluminescent substrate solution ECL (GE Healthcare)

### Quantification and Statistical Analysis

#### Microarray Analysis of Stimulated BMDMs

Expression profiles of M-CSF differentiated BMDMs stimulated for 3 h with either TSBt (1/10), SN*Hh*t (1/10) or Pam3CSK4 (75 ng/ml) were obtained using Illumina MouseWG-6-V2 microarrays (n = 5 for each condition). Probes were used for downstream processing if they were expressed significantly above background (detection P value < 0.05) in at least three samples. This resulted in the analysis of 16, 692 probes (out of a total of 45, 289). Array signal intensities were background adjusted, transformed using the variance-stabilizing transformation and quantile normalized using Lumi from R/Bioconductor. Differential expression analysis was performed for each condition contrast using the empirical Bayes method in LIMMA (Linear Models for Microarray and RNA-seq Data from R/Bioconductor). Significance was defined as a Benjamini-Hochberg adjusted P value < 0.05 and fold change > 2. The union of significantly different probes was visualized in a heatmap and probes were clustered into distinct sets using k-means clustering (k = 4) as implemented in R (k means function in R3.1.0). Sequencing data was generated by the High-Throughput Genomics Group at the Wellcome Trust Centre for Human Genetics (funded by Wellcome Trust grant reference 090532/Z/09/Z).

#### Transcription Factor Motif Enrichment Analysis

We tested for the enrichment of transcription factor motifs among genes that were observed to be specifically upregulated in either SN*Hh*t or Pam3CSK4 conditions. Transcription factor motif annotations were downloaded from the Molecular Signatures Database (MSigDB, C3, motif gene sets at http://software.broadinstitute.org/gsea/msigdb/collections.jsp). These comprise sets of genes that are predicted to be bound by a given transcription factor based on the presence of a conserved motif in their promoter or 3′ UTR. As we were interested in motifs that could readily be attributed to a particular transcription factor, we filtered the database to retain only those motifs that had a designated Transfac (http://www.gene-regulation.com/pub/databases.html) identifier. This resulted in the analysis of 499 motifs (from a total of 836 in the full database). Enrichment analysis was performed using the hypergeometric test implemented in runGO.py from the Computational Genomics Analysis Toolkit (CGAT; https://github.com/CGATOxford/cgat) and gene sets were considered significant at an empirically derived adjusted p value < 0.05.

#### Statistical Analysis

Statistical tests were performed using GraphPad Prism 7.02 and specified in figure legends. Differences were considered to be significant when p < 0.05. All bar charts represent means ± SD.

### Data and Software Availability

#### Accession Numbers/Data Availability Statement

The accession number for the microarray data reported in this paper is ArrayExpress: E-MTAB-5780.

## Supplemental Information

Document S1. Figures S1–S6

Document S2. Article plus Supplemental Information
