# Supplementary material for: A Large Polysaccharide Produced by Helicobacter hepaticus Induces an Anti-inflammatory Gene Signature in Macrophages
Source: Cell Host Microbe. 2017 Dec 13;22(6):733–745.e5. doi: 10.1016/j.chom.2017.11.002 (PMC5734933; doi:10.1016/j.chom.2017.11.002)
Supplement: Document S1. Figures S1–S6 [file mmc1.pdf]

Cell Host & Microbe, Volume *22*

Supplemental Information

A Large Polysaccharide Produced by *Helicobacter hepaticus* Induces an Anti-inflammatory Gene Signature in Macrophages

Camille Danne, Grigory Ryzhakov, Maria Martínez-López, Nicholas Edward Ilott, Fanny Franchini, Fiona Cuskin, Elisabeth C. Lowe, Samuel J. Bullers, J. Simon C. Arthur, and Fiona Powrie

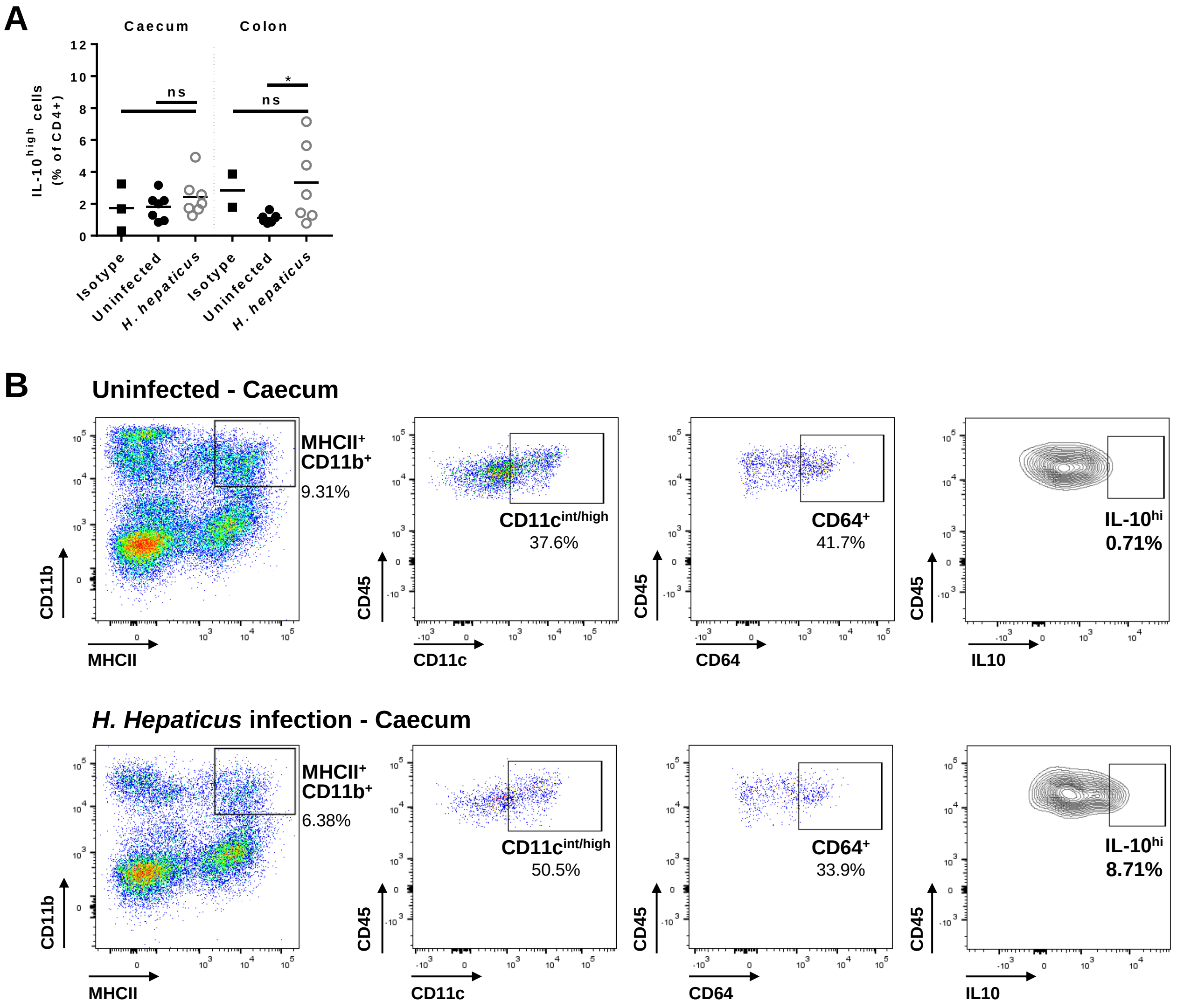

Figure S1. *H. hepaticus* induces IL-10 in gut resident macrophages but not in T cells. Related to Figure 1. (A) Frequency of IL-10^{high} cells among total CD4^{+} T cells in caecum and colon LPL after *H. hepaticus* infection, using an APC anti-mouse IL-10 antibody (clone JES5-16E3) or its isotype control (Rat IgG2b, κ). Mann-Whitney test, $p<0.05$. (B) Gating of MHCII^{+}CD11b^{+}CD11c$^{int/hi}$CD64^{+}IL-10^{high} cells showing one representative sample of the uninfected and *H. hepaticus*-infected caecum groups. Percentages of parent population are indicated.

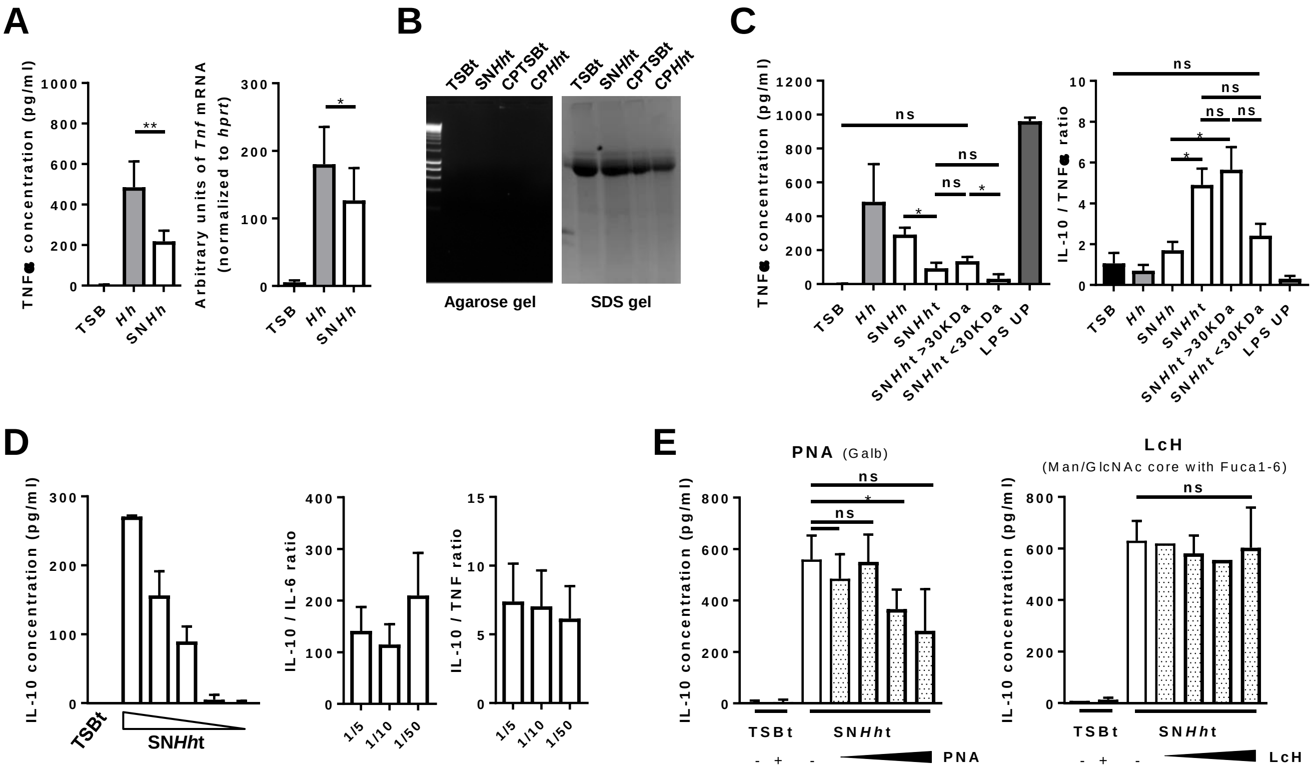

Figure S2. *H. hepaticus* produces a large soluble polysaccharide inducing high IL-10 but low TNFa production in macrophages. Related to Figure 2. (A) mRNA and protein induction of the cytokine TNFa after stimulation with control medium (TSB), *H. hepaticus* whole bacteria (*Hh*) or *H. hepaticus* filtered cultured supernatant (SN*Hh*). (B) Agarose and SDS-page gels of the samples after enzymatic treatment and boiling (TSBt, control medium; SN*Hh*t, treated *H. hepaticus* supernatant) and cold-ethanol precipitation (CPTSBt, crude polysaccharide fraction of TSBt; CP*Hh*t, crude polysaccharide fraction of SN*Hh*t). The agarose gel shows no contaminating DNA. The SDS-page gel shows one band present in all samples that corresponds to the proteinase K used to treat the samples, and no other significant protein contamination. (C) Induction of TNFa and IL-10/TNFa ratio after a 3 h stimulation with SN*Hh* treated with a combination of DNAse, RNAse and proteinase K and boiled (2 h, 95 °C) (SN*Hh*t), SN*Hh*t fractionated by size using 30KDa Vivaspin columns (SN*Hh*t>30KDa and SN*Hh*t<30KDa), and LPS UP. (D) Dose-dependent induction of IL-10 after a 3 h stimulation with SN*Hh*t and the calculated IL-10/ IL-6 and IL-10/TNFa ratios. (E) Induction of IL-10 after 3 h stimulation with TSBt, LPS or SN*Hh*t depleted using various v/v concentrations of Peanut agglutinin (PNA) or Lentil (LcH)-coated lectin beads. Data from three independent experiments. Mann-Whitney test, $p<0.05$. Mean ± SD.

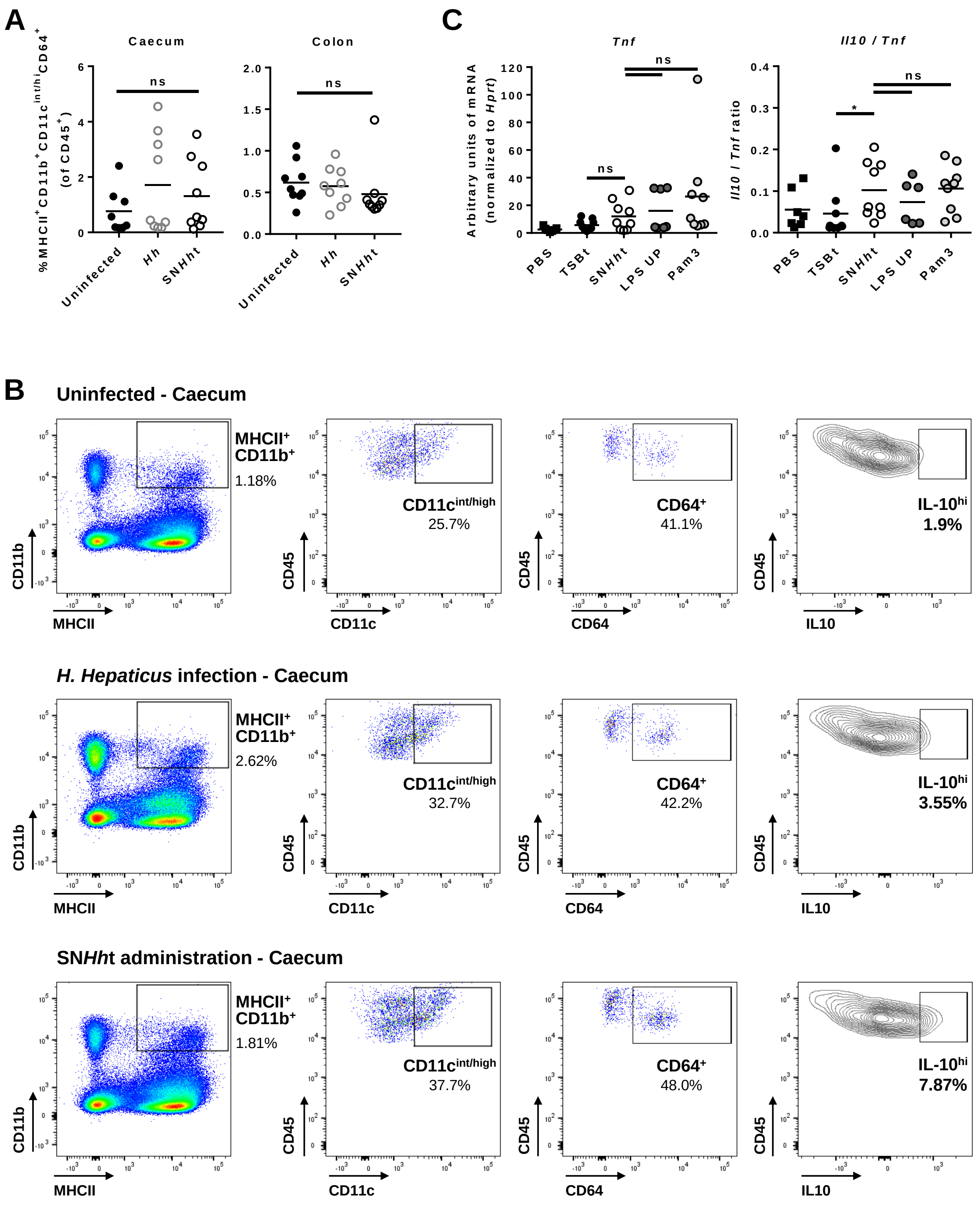

Figure S3. *H. hepaticus* polysaccharide does not increase the frequency of lamina-propria resident macrophages and induces limited amount of TNFa when injected intraperitoneally. Related to Figure 3. (A) Frequency of resident macrophages among total $CD45^+$ cells from caecum and colon LPL. SPF WT mice were infected with *Hh*, or orally gavaged with TSBt or SN*Hh*t for 3 days. (B) Gating of $MHCII^+CD11b^+CD11c^{int/hi}CD64^+IL\text{-}10^{high}$ cells showing one representative sample of caecum from mice infected or not with *H. hepaticus* or after administration of SN*Hh*t. Percentages of parent population are indicated. (C) *tnf* mRNA expression levels and *Il10/Tnf* ratio in the peritoneal cell fraction after 6 h challenge. Each stimulus (TSBt and SN*Hh*t, 200 ml; LPS UP and Pam3, 50 mg) were injected in the intraperitoneal cavity of SPF WT mice. Each symbol represents an individual mouse (three independent experiments). Mann-Whitney test, $p<0.05$.

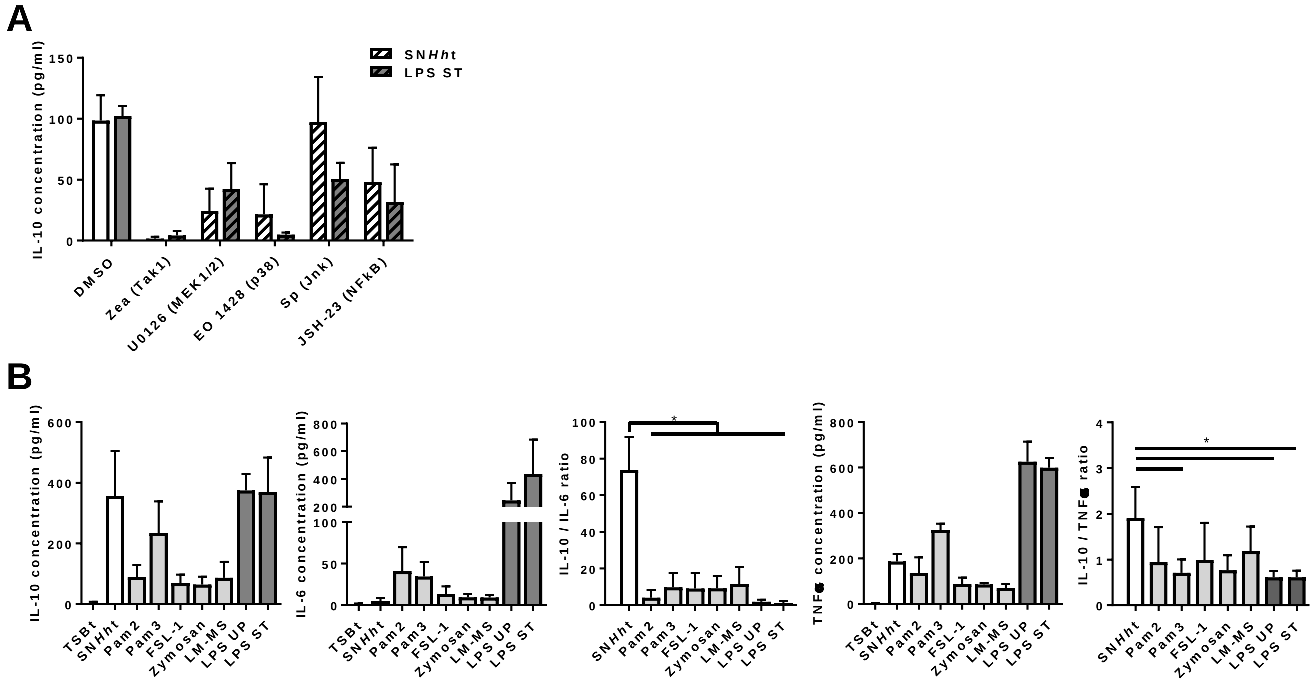

Figure S4. *H. hepaticus* polysaccharide signals through the classical TLR2/MyD88 pathway but triggers higher anti-inflammatory ratios compared to canonical TLR2 ligands. Related to Figure 4. (A) Induction of IL-10 production in WT BMDMs pre-treated for 30 min with a kinase inhibitor or DMSO prior stimulation for 3 h with SN*Hh*t or LPS ST. Zea (5z-7-oxozeanol, Tak1) at 1 mM, UO126 (MEK1/2), JSH-23 (NF-kb) and Sp600125 (JNK) at used at 10 mM and EO 1428 (p38) at 5 mM. (B) IL-10, IL-6 and TNFa production, and IL-10/IL-6 and IL-10/TNFa protein ratios induced by various TLR2 and TLR4 ligands after 3 h stimulation. Pam2 (Pam2CSK4, TLR2/6 ligand); Pam3 (Pam3CSK4, TLR2/1 ligand); FSL-1 (TLR2/6 ligand); Zymosan, cell wall from *Saccharomyces cerevisiae* (TLR2 ligand); LM-MS, Lipomannan from *Mycobacterium smegmatis* (TLR2 ligand); LPS UP, LPS ultrapure from *E. coli* O111:B4 (TLR4 ligand); LPS ST, LPS standard from *E. coli* O55:B5 (TLR4 and TLR2 ligand). Data from three independent experiments. Mann-Whitney test, $p<0.05$. Mean ± SD.

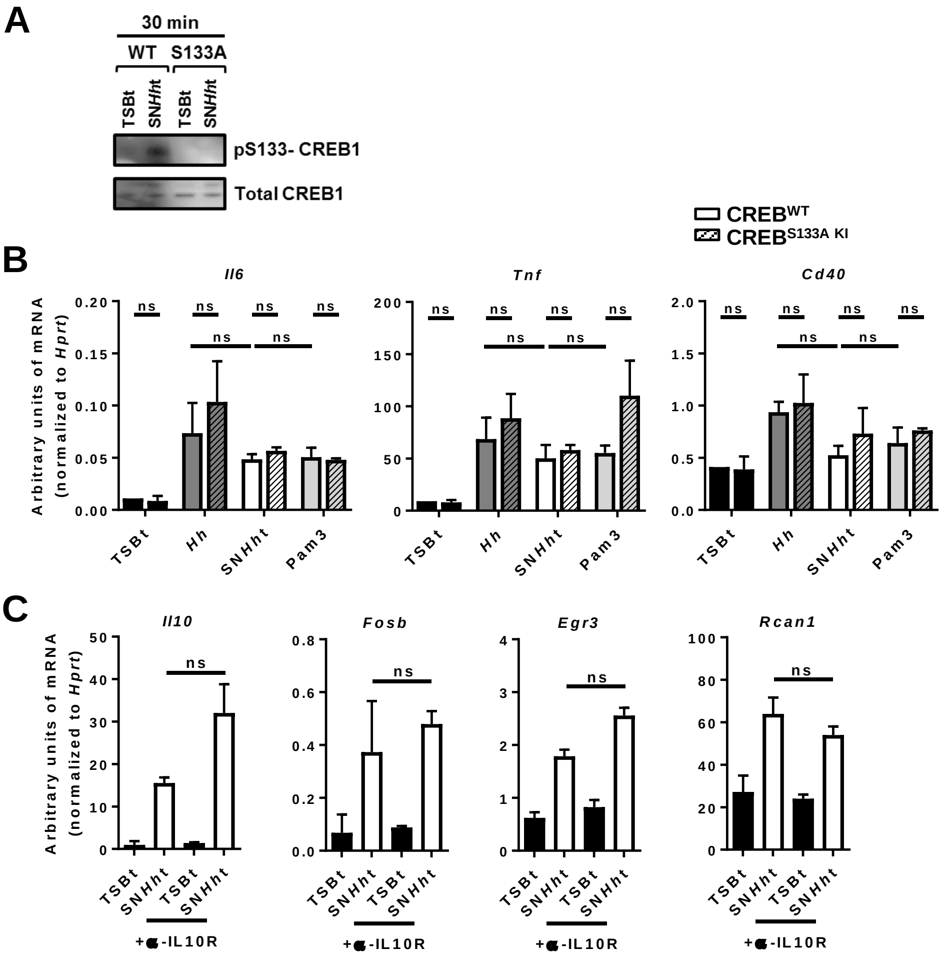

Figure S5. The induction of CREB target genes by *H. hepaticus* polysaccharide is not dependent on IL-10R signaling. Related to Figure 6. (A) Western Blot showing the absence of CREB1 S133 phosphorylation in BMDMs from conditional CREB$^{S133A\ KI}$ *vav*-cre+ mice after stimulation for 30 min with TSBt or SN*Hh*t. (B) mRNA levels of *Il6, Tnf* and *Cd40* genes in BMDMs from conditional CREB^{WT} and CREB$^{S133A\ KI}$ *vav*-cre+ mice after stimulation for 1 h with TSBt, *Hh*, SN*Hh*t or Pam3 (one of three independent experiments). Two-way ANOVA and Sidak's and Tukey's multiple comparisons tests, $p<0.05$. (C) Induction of *Il10, Fosb, Egr3*, and *Rcan1* mRNA expression in WT BMDMs pre-treated for 2 h with a polyclonal anti-IL-10R blocking antibody (anti-IL-10R) before stimulation with TSBt or SN*Hh*t for 3 h. One of three independent experiments. Mann-Whitney test, $p<0.05$. Mean ± SD.

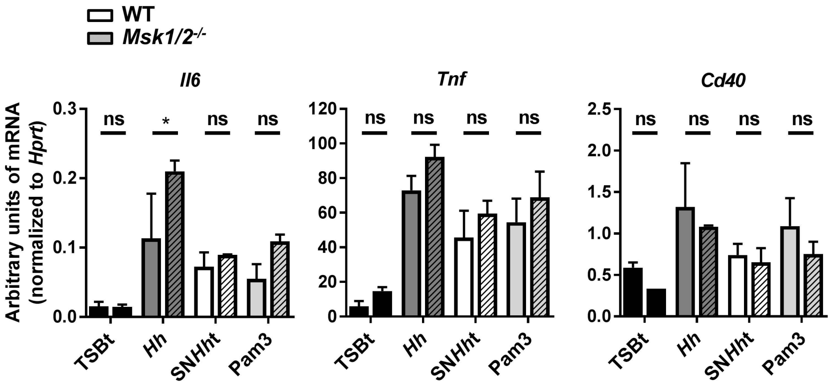

Figure S6. Induction of MSK1/2-independent genes by *H. hepaticus* polysaccharide. Related to Figure 7. mRNA levels of *Il6, Tnf, Cd40* genes in BMDMs from WT or MSK1/2$^{-/-}$ mice after stimulation for 1 h with TSBt, *Hh*, SN*Hh*t or Pam3. One of three independent experiments. Two-way ANOVA and Sidak's multiple comparisons test, $p<0.05$. Mean ± SD.
